# Membrane-Tethered Mucin 1 Is Stimulated by Interferon and Virus Infection in Multiple Cell Types and Inhibits Influenza A Virus Infection in Human Airway Epithelium

**DOI:** 10.1128/mbio.01055-22

**Published:** 2022-06-14

**Authors:** Ethan Iverson, Kira Griswold, Daniel Song, Talita B. Gagliardi, Kajal Hamidzadeh, Mehmet Kesimer, Sanju Sinha, Melissa Perry, Gregg A. Duncan, Margaret A. Scull

**Affiliations:** a Department of Cell Biology & Molecular Genetics, Maryland Pathogen Research Institute, University of Maryland, College Park, Maryland, USA; b Fischell Department of Bioengineering, University of Maryland, College Park, Maryland, USA; c Marsico Lung Institute, University of North Carolina, Chapel Hill, North Carolina, USA; d Cancer Data Science Laboratory, National Cancer Institute, National Institutes of Health, Bethesda, Maryland, USA; e Center for Bioinformatics and Computational Biology, University of Maryland, College Park, Maryland, USA; St. Jude Children's Research Hospital; Johns Hopkins Bloomberg School of Public Health

**Keywords:** influenza virus, mucin, interferon, airway epithelium, macrophage

## Abstract

Influenza A virus (IAV) causes significant morbidity and mortality in the human population. Tethered mucin 1 (MUC1) is highly expressed in airway epithelium, the primary site of IAV replication, and also by other cell types that influence IAV infection, including macrophages. MUC1 has the potential to influence infection dynamics through physical interactions and/or signaling activity, yet MUC1 modulation and its impact during viral pathogenesis remain unclear. Thus, we investigated MUC1-IAV interactions in an *in vitro* model of human airway epithelium (HAE). Our data indicate that a recombinant IAV hemagglutinin (H3) and H3N2 virus can bind endogenous HAE MUC1. Notably, infection of HAE with H1N1 or H3N2 IAV strains does not trigger MUC1 shedding but instead stimulates an increase in cell-associated MUC1 protein. We observed a similar increase after type I or III interferon (IFN) stimulation; however, inhibition of IFN signaling during H1N1 infection only partially abrogated this increase, indicating that multiple soluble factors contribute to MUC1 upregulation during the antiviral response. In addition to HAE, primary human monocyte-derived macrophages also upregulated MUC1 protein in response to IFN treatment and conditioned media from IAV-infected HAE. Then, to determine the impact of MUC1 on IAV pathogenesis, we developed HAE genetically depleted of MUC1 and found that MUC1 knockout cultures exhibited enhanced viral growth compared to control cultures for several IAV strains. Together, our data support a model whereby MUC1 inhibits productive uptake of IAV in HAE. Infection then stimulates MUC1 expression on multiple cell types through IFN-dependent and -independent mechanisms that further impact infection dynamics.

## INTRODUCTION

The respiratory epithelium encodes large and extensively glycosylated proteins, termed mucins, to maintain airway surface hydration and protect the underlying cells from environmental insults, such as respiratory viruses (1, 2). While some mucins are secreted and form a mucus gel, others—the aptly named “tethered” mucins—remain anchored to the apical epithelial cell surface, giving rise to the periciliary layer (PCL) (1–3). The PCL serves as a platform for overlying secreted mucins, allowing ciliary action to propel the secreted mucus gel in a process known as mucociliary clearance (MCC) (4, 5). Additionally, tethered mucins of the PCL represent steric obstacles to impede further access to the underlying epithelium (2). In addition to the bulky extracellular domain (ED) typical of tethered mucins, the highly abundant mucin 1 (MUC1) features a well-conserved cytoplasmic tail (CT) that can be differentially phosphorylated (6, 7) and interact with many partners, including kinases and adapter proteins involved in signal transduction (3, 8, 9). The presence of an autoproteolytic SEA domain upstream of the transmembrane domain, in conjunction with enzymatic sheddases, can lead to the release of the MUC1-ED domain from the MUC1-CT domain (3, 10). MUC1-CT can also be translocated to the nucleus (11–13), supporting important functions outside its canonical representation among the PCL.

MUC1/Muc1 (humans/mice) has been implicated in various aspects of both bacterial and viral infections. For example, the genetic disruption of *Muc1* is associated with elevated inflammation and faster Pseudomonas aeruginosa clearance (8) yet results in more severe Streptococcus pneumoniae infection (14). Adenoviral infection in *Muc1^−/−^* mice is modestly increased with no significant inflammatory differences in the lung (15), and adenoviral vector gene transfer efficiency *in vitro* and *in vivo* is inhibited by MUC1/Muc1 expression (16, 17), suggesting that MUC1 restricts adenovirus by acting as a physical barrier. Outside the airway, MUC1 has been shown to be an attachment factor for Helicobacter pylori (18) and Salmonella enterica (19), while the presence of MUC1 in breast milk is protective against human immunodeficiency virus transmission (20). MUC1 has also been shown to suppress respiratory syncytial virus-induced inflammation *in vitro* by forming a negative feedback loop with tumor necrosis factor alpha (TNF-α) (21), and altered expression of MUC1 has been described in response to multiple inflammatory stimuli (22), suggesting that it might play a universal and dynamic role during insult by different pathogens (23, 24). Notably, no consensus on MUC1 function or dynamics during infection is reflected in these studies.

Influenza A virus (IAV) infects the human airway epithelium (HAE) (25) and causes an estimated annual burden of 290,000 to 645,000 deaths worldwide in nonpandemic years (26). To gain access to airway epithelial cells, IAV must first penetrate the secreted mucus and underlying PCL barriers. Subsequent endocytic uptake into epithelial cells is mediated through interactions between the viral attachment protein hemagglutinin (HA) and glycans with terminal sialic acid (SA) linkages on the cell surface (27). While it is known that SA recognition heavily impacts cellular tropism and epizootic potential (28), the extent of IAV attachment to SA and consequences for specific host proteins are unclear (29). A recent report suggests that IAV can interact with the extracellular domain of MUC1 and that this interaction has important implications for pathogenesis *in vivo* (30). However, it is not known if MUC1 can restrict IAV access to well-differentiated epithelial cells or if SA-mediated interactions subvert a normally protective physical role and instead support IAV uptake. Additionally, it is not known how MUC1 expression is impacted during IAV infection of the respiratory epithelium and whether its immunomodulatory role is important in the context of IAV pathogenesis.

Here, we investigated specific interactions between IAV and MUC1 in a physiologically relevant model of HAE. Consistent with previous reports in cell lines (30), we show that IAV can interact with membrane-tethered MUC1 in HAE; however, in contrast to earlier findings, we found no evidence of IAV-mediated MUC1 shedding in several epithelial model systems. Our data instead indicate that MUC1 is upregulated in all HAE component cell types as well as primary human monocyte-derived (PMD) macrophages by soluble factors, including type I and type III interferons, produced during IAV infection. Then, using an *in vitro* HAE model system with a genetic deletion of MUC1, we demonstrate that depletion of MUC1 is proviral for several IAV strains, leading to enhanced IAV replication and spread.

## RESULTS

### The IAV hemagglutinin protein binds MUC1 isolated from HAE apical secretions and colocalizes with MUC1 during infection.

**FIG 1:**
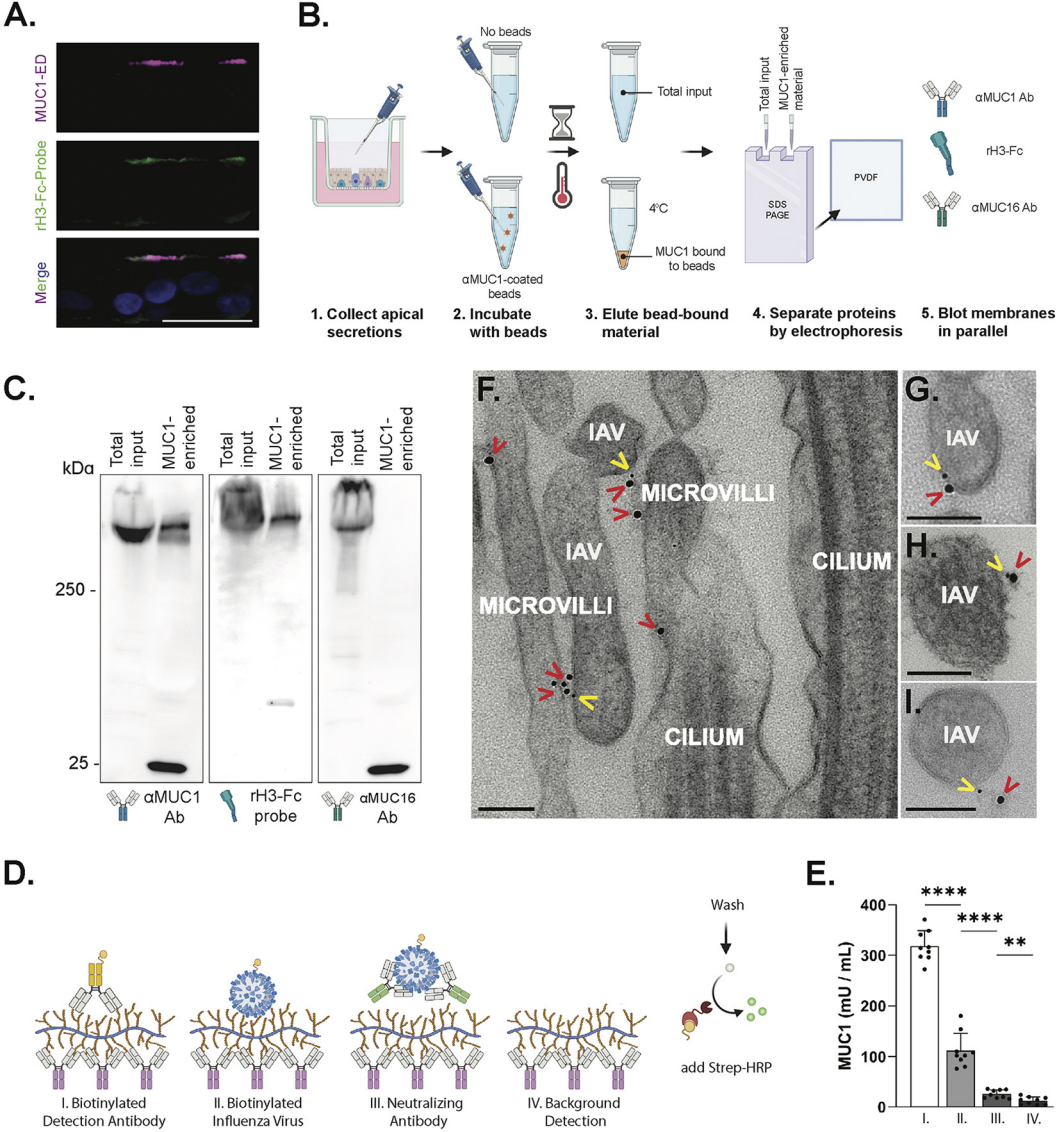
IAV binds HAE-derived MUC1. (A) Cross-section of normal HAE, immunostained for the extracellular domain of MUC1 (MUC1-ED, purple) and with a trimerized hemagglutinin probe (rH3-Fc-Probe, green). The merged channel includes nuclei (blue). Bar = 20 μm. (B) Schematic diagram of MUC1 enrichment from HAE and analysis, showing HAE apical wash collection, immunoprecipitation of MUC1-containing material, gel-electrophoretic separation of bead-bound material, and subsequent Western blot probes used for panel C. (C) Crude HAE wash (total input) and MUC1-IP material (MUC1-enriched) were separated by SDS-PAGE in triplicate and probed in parallel, as indicated. Lower bands in the MUC1-enriched lanes indicate secondary antibody-mediated detection of heavy and light antibody chains used in the immunoprecipitation. (D) Cartoon schematic of the modified MUC1 ELISA protocol. MUC1 immobilized in the ELISA well by the anti-MUC1 capture antibody (purple Ig) was subsequently incubated with biotinylated anti-MUC1 detection antibody (I; yellow, Ig), biotinylated Udorn (II), or biotinylated Udorn mixed with neutralizing anti-influenza virus HA antibody (III, green, Ig) as indicated. Binding was detected through the addition of Strep-HRP, and corresponding ELISA results are shown in panel E. (E) Data obtained from three experiments, utilizing three different HAE donors, with three biological replicates per donor. (F to I) Transmission electron microscopy of HAE after adsorption with A/Udorn/307/72 (H3N2) influenza virus. Images were taken from primary HAE (F, G, and I) and an immortalized HAE cell line (BCi-NS1.1) (H). MUC1 (red carets) and H3 (yellow carets) were detected with 18-nm- and 6-nm-gold-nanoparticle-conjugated antibodies, respectively. Bars = 100 nm. Experimental results were analyzed by the Mann-Whitney U test between indicated conditions. All data are significant where indicated (**, *P* < 0.01; ****, *P* < 0.0001). Diagrams in panels B and D were created with BioRender.com.

Previous work suggests that IAV can interact with MUC1 based on fluorescence microscopy and colocalization analysis in A549 cells (30). Thus, we sought to determine if the IAV HA protein binds MUC1 derived from an *in vitro* model of primary HAE, since this system recapitulates important aspects of airway epithelial morphology and physiology, including both secreted and tethered mucin expression (Fig. S1) (2, 31, 32). In support of a potential HA-MUC1 interaction in HAE, our initial experiments revealed recombinant, Fc-tagged H3 hemagglutinin (rH3-Fc) binding at the apical cell surface in histological cross sections of HAE cultures in regions that also stained positive for MUC1-ED (Fig. 1A).

10.1128/mbio.01055-22.1 FIG S1 The HAE system recapitulates airway epithelial morphology and tethered mucin expression. Immunohistochemistry of primary HAE cultures detecting the extracellular domains of tethered mucins MUC1, MUC4, and MUC16 (green; nuclei, blue). MUC4 and MUC16 stains represent immature glycosylation forms, while mature proteins localize to the extracellular apical lumen. Bar = 20 μm. Download FIG S1, TIF file, 1.4 MB. Copyright © 2022 Iverson et al. 2022 Iverson et al. https://creativecommons.org/licenses/by/4.0/ This content is distributed under the terms of the Creative Commons Attribution 4.0 International license.

Notably, in HAE cultures, MUC1 can also be identified in apical secretions along with other, less abundant tethered mucins (e.g., MUC4 and MUC16) (2, 33); thus, to further interrogate HA-MUC1 interaction, we enriched for MUC1 in HAE secretions by immunoprecipitation with anti-MUC1-coated beads (Fig. 1B). This MUC1-enriched material was then washed and eluted off the beads before being separated by SDS-PAGE and transferred to a membrane, where interaction with influenza virus hemagglutinin protein was determined using rH3-Fc as a probe. Detection of rH3-Fc binding and anti-MUC1-ED reactivity in the same region of the membrane indicated a likely interaction between the viral attachment protein and this mucin molecule (Fig. 1C). MUC16, another tethered mucin that was also previously identified in HAE secretions, was detected in the total input but not in the immunoprecipitated conditions. These data support the idea that MUC1 was further enriched from other tethered mucins and are consistent with the conclusion that detection of rH3-Fc is indicative of hemagglutinin-MUC1 binding.

Since the rH3-Fc probe represents a soluble form of HA, we next sought to determine if whole virions could interact with HAE-derived MUC1. Toward this goal, we utilized sulfo-NHS(N-Hydroxysulfosuccinimide)-SS-biotin to label sucrose-purified A/Udorn/307/72, a well-characterized strain that natively possesses a H3 similar to the recombinant H3 probe, and confirmed that the labeled virus (Udorn^biotin^) retained infectivity in HAE (Fig. S2). We then asked whether Udorn^biotin^ could interact with MUC1 using a modified enzyme-linked immunosorbent assay (ELISA) scheme (Fig. 1D). Here, MUC1 in HAE culture lysates was isolated by MUC1 capture antibody coating the bottom of the ELISA plate. Subsequent binding of 3 × 10^4^ PFU of Udorn^biotin^ or biotinylated-anti-MUC1 detection antibody (provided in the ELISA kit and used as a positive control) to the immobilized MUC1 in the well was assessed using horseradish peroxidase-conjugated streptavidin (Strep-HRP) and the addition of HRP substrate. To control for interactions between Udorn^biotin^ and MUC1 not mediated through the viral HA, we ran a parallel condition in which Udorn^biotin^ was incubated in the ELISA well in the presence of large amounts of neutralizing anti-H3 goat serum shown to abolish all hemadsorption activity of the biotinylated virus (E. Iverson and M. A. Scull, unpublished data). Udorn^biotin^ was able to support the detection of MUC1 primarily through interactions with HA, as indicated by the nearly complete loss of reactivity after incubation with neutralizing antibodies (Fig. 1E). Together with our data in Fig. 1C, these results indicate that the hemagglutinin of IAV can mediate interactions with purified forms of HAE-derived MUC1.

10.1128/mbio.01055-22.2 FIG S2 Biotinylation of IAV has minimal impact on viral titer and tropism. (A)Equal amounts of A/Udorn/307/72 were either biotinylated with sulfo-NHS-SS-biotin or treated with DMSO as a vehicle control prior to analysis by nonreducing Western blotting. Samples were probed with anti-HA and streptavidin-HRP. (B) Labeled Udorn and vehicle control were analyzed by plaque assay to confirm retention of infectivity following biotinylation treatment. (C) HAE were infected with biotinylated and vehicle-treated control Udorn prior to fixation and *en face* immunostaining. Cells positive for a ciliated cell marker (acetylated alpha-tubulin) are indicated in red; cells positive for viral antigen (nucleoprotein) are indicated in green. Download FIG S2, TIF file, 2.6 MB. Copyright © 2022 Iverson et al. 2022 Iverson et al. https://creativecommons.org/licenses/by/4.0/ This content is distributed under the terms of the Creative Commons Attribution 4.0 International license.

Finally, to determine if the influenza virus-MUC1 interaction occurs during infection in the context of the native HAE microenvironment, we inoculated HAE cultures with >5 × 10^5^ PFU (approximate multiplicity of infection [MOI] of 10) of A/Udorn/307/72 and subsequently chilled the cultures to 4°C so as to irreversibly stabilize virus adsorption and restrict cellular entry (34). Next, we performed transmission electron microscopy with immunogold labeling to detect IAV H3 as well as MUC1-ED, allowing us to observe potential colocalization of these two molecules prior to cellular uptake. MUC1 was once again identified at the apical surface, primarily localized to microvilli, as previously described (2). IAV was also found in close proximity to immunogold-labeled MUC1 (Fig. 1F to I), in line with our *in vitro* interactions and prior work in A549 cells (30). Taken together, our results suggest that influenza virus interacts with MUC1 during the early stages of infection in a physiologically relevant system that recapitulates the extracellular environment in the airway.

### IAV replication in HAE is not associated with an increase in soluble MUC1.

**FIG 2:**
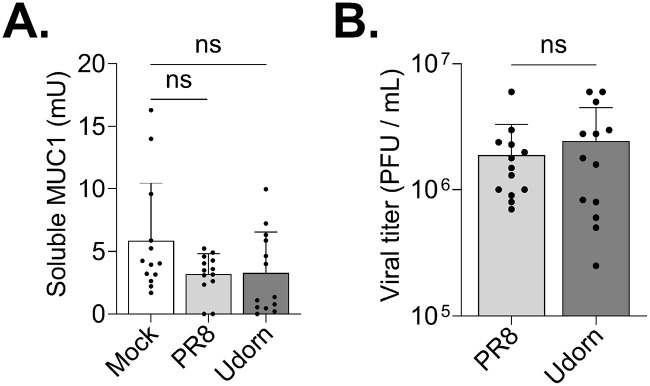
IAV replication in HAE is not associated with an increase in soluble MUC1. HAE cultures were infected with 5 × 10^4^ PFU (approximate MOI of 1) of either A/PR/8/34 or A/Udorn/307/72 or mock infected. After 24 h, apical HAE compartments were washed with PBS, which was used to determine (A) soluble MUC1-ED by ELISA and (B) viral titer by plaque assay. Data were obtained from four experiments, utilizing three different HAE donors, with at least three biological replicates per donor. Experimental results were analyzed by the Mann-Whitney U test compared to mock conditions or each other (ns, not significant).

Given our results indicating HAE-MUC1 interacts with IAV hemagglutinin, we next sought to determine the consequence of this interaction. Previous work in CHO cells suggested that the ectodomain of ectopically expressed MUC1 could act as a releasable decoy that is shed upon IAV binding to prevent subsequent infection of underlying cells (30). To determine whether MUC1 is shed during viral challenge in the context of the airway PCL, we inoculated primary, well-differentiated HAE cultures with either A/Udorn/307/72 or another well-characterized virus, A/PR/8/34, which possesses a H1 hemagglutinin, and quantified MUC1 and infectious virus in apical washes 24 h postinfection (hpi) (Fig. 2). Surprisingly, in contrast to previous observations, we found that neither A/PR/8/34 nor A/Udorn/307/72 infection resulted in a significant change in soluble MUC1 levels relative to mock-infected cultures despite reaching mean titers of 1.9 × 10^6^ and 2.4 × 10^6^ PFU/ml, respectively.

To determine if a lack of MUC1 shedding after IAV challenge was an HAE-specific phenomenon, we executed a similar experiment in A549 cells expressing endogenous MUC1. Following a 1-h incubation with an MOI of 1 at 4°C to allow viral particles to bind to the cell surface, we removed the inoculum, returned the cultures to 37°C, and quantified MUC1 and infectious virus in cell culture supernatants 24 h later (Fig. S3). Similar to our HAE results, infection in A549 cells with A/PR/8/34 or A/Udorn/307/72 did not trigger an increase in MUC1 shedding; in fact, a significant decrease in soluble MUC1 was observed following inoculation with A/Udorn/307/72. These data corroborate our results in HAE and together suggest that MUC1 expressed endogenously in human airway cells is not shed during IAV challenge.

10.1128/mbio.01055-22.3 FIG S3 A549 cells express MUC1 at baseline but do not shed MUC1 following IAV infection. (A) A549 cells were stained for the extracellular domain of MUC1 and nuclei. Bar = 50 μm. (B) A549 cells were infected with IAV as indicated, and MUC1 in the cell culture supernatants 24 hpi was quantified by ELISA. Data in panel B are from four experimental replicates. Data were analyzed by the Mann-Whitney U test compared to mock conditions (ns, not significant; **, *P* < 0.01). Download FIG S3, TIF file, 1.3 MB. Copyright © 2022 Iverson et al. 2022 Iverson et al. https://creativecommons.org/licenses/by/4.0/ This content is distributed under the terms of the Creative Commons Attribution 4.0 International license.

### Cell-associated MUC1 levels are upregulated during IAV infection and after interferon treatment.

**FIG 3:**
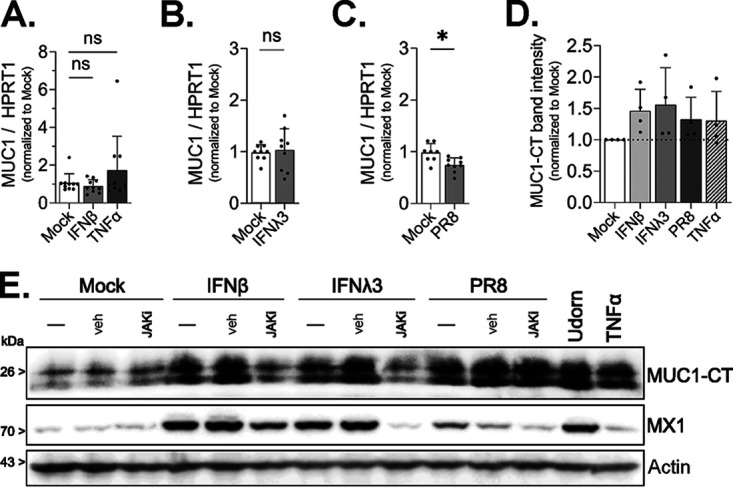
Cell-associated MUC1 levels are upregulated during IAV infection and IFN treatment. HAE were stimulated with (A) IFN-β, (A) TNF-α, or (B) IFN-λ3 or (C) infected with PR8 (5 × 10^4^ PFU; approximate MOI of 1), and MUC1 expression was quantified by qPCR after 24 h of treatment. (D) HAE were stimulated as indicated or infected with PR8 as for panels A to C for 24 h, protein lysate collected, and MUC1 expression quantified by Western blotting for MUC1-CT. MUC1-CT band intensity was analyzed by densitometry relative to actin band intensity. (E) HAE were stimulated with IFN-β, IFN-λ3, or PR8 alone (–) or in the presence of the JAK inhibitor ruxolitinib (JAKi) or DMSO as a vehicle control (veh). Additional cultures were stimulated with Udorn or TNF-α alone. After 24 h, lysate was collected and analyzed by Western blotting for MUC1-CT, MX1, or actin. Results in panels A to C are from three experimental replicates utilizing three different HAE donors with a minimum of three biological replicates from each donor. The densitometry analysis (D) shows data from four experimental replicates utilizing four different HAE donors with one biological replicate from each donor. All experimental results were analyzed by the Mann-Whitney U test compared to mock conditions and are significant where indicated (*, *P* < 0.05; ns, not significant).

As the lack of an increase in soluble MUC1 levels following infection of human airway cells was unexpected, we sought to further characterize MUC1 dynamics in HAE after IAV challenge. Since previous reports have described an increase in MUC1 protein following gamma interferon (IFN-γ) exposure in other systems (35), and IAV infection of HAE triggers both type I and type III IFN (36), we quantified MUC1 gene expression and cell-associated MUC1 protein levels following IAV infection, or after treatment of HAE with IFN-β, IFN-λ3, or TNF-α (previously implicated in upregulating MUC1 [23, 37]). Neither type I or type III IFN treatment (Fig. 3A and B) nor A/PR/8/34 infection (Fig. 3C) triggered an increase in MUC1 transcripts above mock-treated controls, let alone a response typical of well-characterized interferon-stimulated genes (Fig. S4). However, type I and type III IFN, along with IAV, were able to stimulate production of MUC1 protein similar to that seen with TNF-α (Fig. 3D). Furthermore, IAV-mediated upregulation of MUC1 protein was at least partially IFN signaling independent, as the addition of a Janus tyrosine kinase 1 (JAK1) inhibitor did not abolish increased MUC1 protein expression as it did for MX1, a marker for IFN signaling (Fig. 3E).

10.1128/mbio.01055-22.4 FIG S4 Expression of well-characterized interferon-stimulated genes and inflammatory chemokines in HAE following cytokine stimulation. HAE were stimulated with recombinant human IFN-β or TNF-α or subjected to mock conditions for 24 h. Total RNA was then collected, and (A) MX1 and (B) IL-8 expression were analyzed by qPCR. (C) HAE were stimulated with recombinant IFN-λ3 for 24 h prior to RNA collection and qPCR quantification of MX1 as before. Data in panels A to C are from three experimental replicates utilizing three different HAE donors with at least three biological replicates from each donor. Data were analyzed by the Mann-Whitney U test compared to mock conditions (ns, not significant; **, *P* < 0.001; ****, *P* < 0.0001). Download FIG S4, TIF file, 0.3 MB. Copyright © 2022 Iverson et al. 2022 Iverson et al. https://creativecommons.org/licenses/by/4.0/ This content is distributed under the terms of the Creative Commons Attribution 4.0 International license.

**FIG 4:**
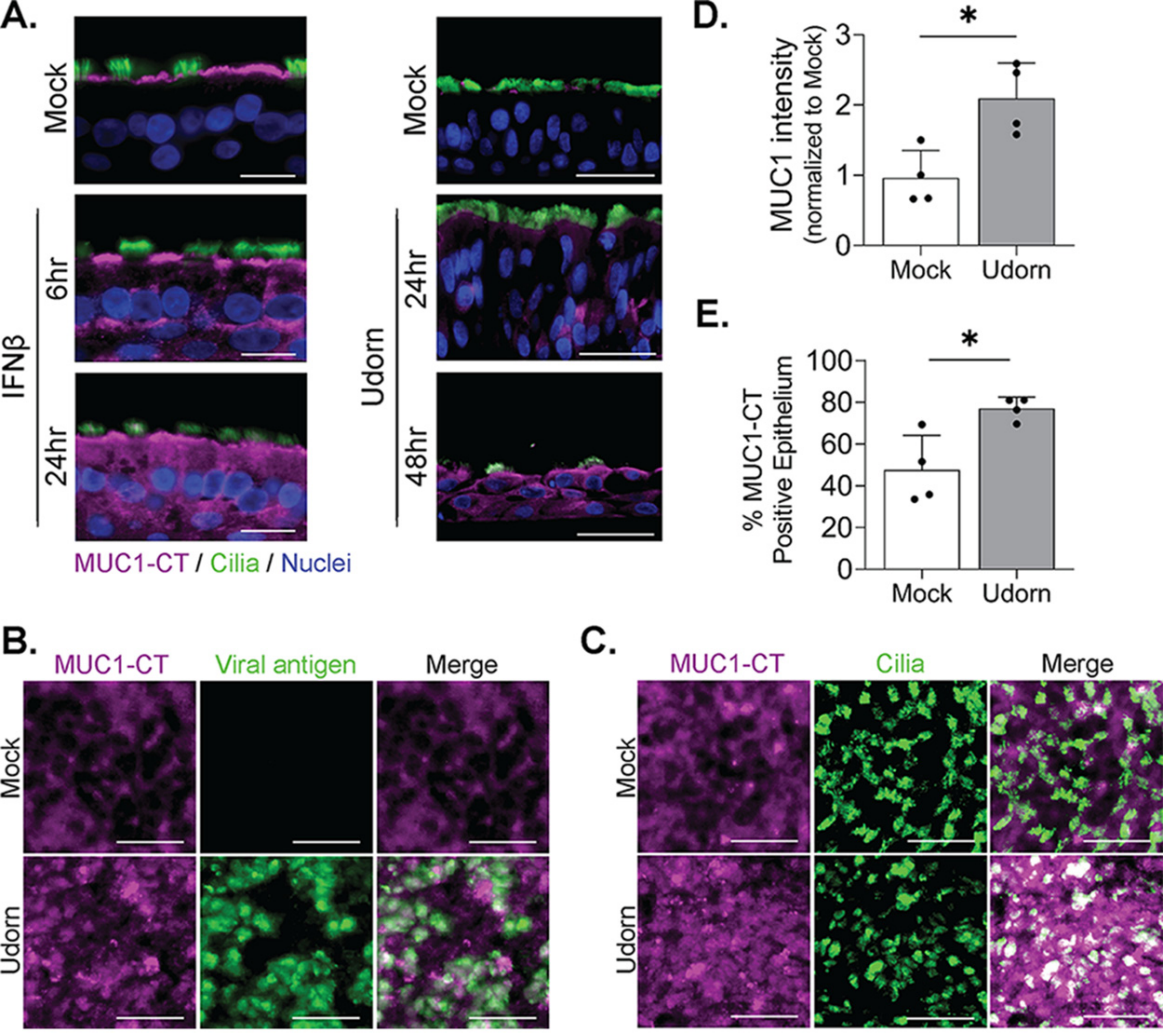
Type 1 IFN and IAV broadly upregulate MUC1 expression across HAE. (A) HAE were stimulated with IFN-β or mock stimulated (left) or infected with Udorn (5 × 10^2^ PFU, approximate MOI of 0.01) (right) and fixed for immunohistochemical detection of MUC1-CT (purple), acetylated alpha-tubulin (cilium marker; green), and nuclei at the indicated time points. (B to E) HAE were infected with Udorn (5 × 10^4^ PFU, approximate MOI of 1), fixed at 24 hpi, and stained *en face* for MUC1-CT (purple) along with (B) viral antigen (nucleoprotein, green), or (C) a ciliated cell marker (acetylated alpha-tubulin; green). The mean intensity (D) or total area staining positive for MUC1-CT (E) was quantified by FIJI on four additional cultures across two donors after infection as for panels B and C and analyzed by the Mann-Whitney U test compared to the mock condition, indicating significance (*, *P* < 0.05). Results in panel A are from one experimental replicate utilizing two different HAE donors (left and right) and one biological replicate from each donor. Results in panels B and C are from the same donor. Results in panels D and E are from two experimental replicates utilizing two different HAE donors with two biological replicates from each donor. Bars = 20 μm (A) and 25 μm (B and C).

In order to visualize which cells expressed MUC1 after IFN challenge or IAV infection, we fixed cultures either 6 and 24 h after IFN treatment or 24 and 48 hpi and stained for MUC1 using standard immunohistochemical approaches. Surprisingly, despite a lack of protein expression in basal cell populations at baseline and a lack of mRNA upregulation after IFN treatment (Fig. S1 and Fig. 3A), we observed MUC1 protein in all HAE component cell types following IFN-β stimulation (Fig. 4A, left). Similarly, infection of HAE with A/Udorn/307/72 (500 PFU; approximate MOI of 0.01) was associated with ubiquitous MUC1 protein expression throughout the epithelium by 48 hpi (Fig. 4A, right). Inoculation of HAE cultures with a higher dose of A/Udorn/307/72 (50,000 PFU; approximate MOI of 1) followed by *en face* immunofluorescence staining 24 hpi (Fig. 4B and C) supported these findings, showing a significant increase in both MUC1 fluorescence intensity (Fig. 4D) and MUC1-positive area (Fig. 4E) across the apical surface compared to uninfected baseline conditions. Together, our data show that MUC1 protein is broadly expressed in HAE after IFN exposure and IAV infection.

### Soluble factors secreted by HAE during IAV infection upregulate MUC1 on primary human monocyte-derived macrophages.

**FIG 5:**
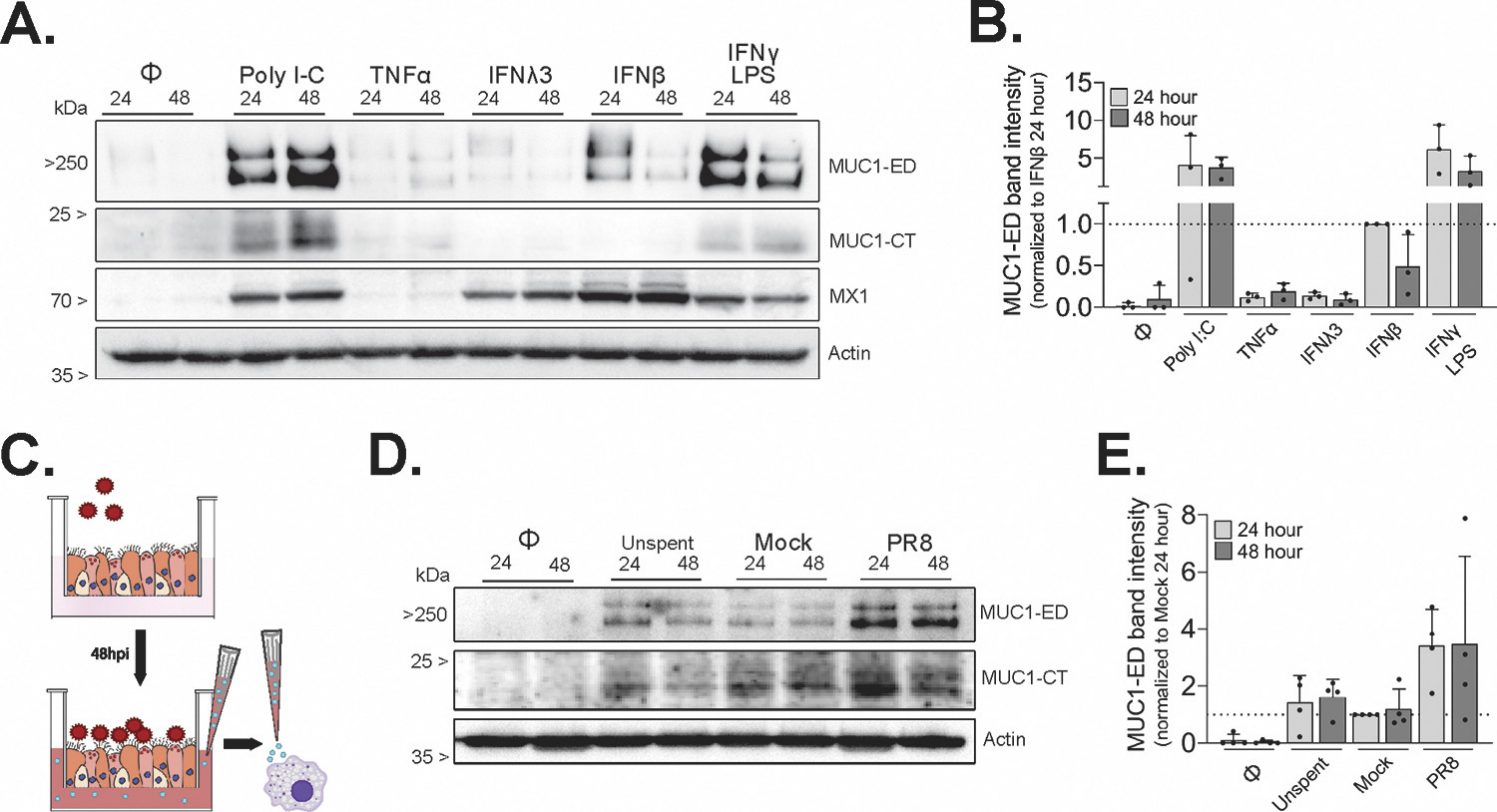
Primary human monocyte-derived macrophages upregulate MUC1 in response to IFN and soluble factors produced from IAV-infected HAE. GM-CSF-derived PMD macrophages were either untreated (Φ) or stimulated as indicated for 24 or 48 h. (A) Cell lysates were then collected and analyzed by Western blotting for MUC1-ED, MUC1-CT, MX1, and actin. (B) Densitometry across conditions used for panel A. Data represent MUC1-ED relative to actin for each sample, normalized to IFN-β/24 h. (C) Cartoon schematic of experiment conditions used for panel D, where PMD macrophages were stimulated for 24 or 48 h with freshly prepared (Unspent), mock-conditioned (Mock), or PR8-infected HAE-conditioned basolateral media (PR8) before lysate collection and Western blot analysis for MUC1-ED, MUC1-CT, and actin. HAE-conditioned basolateral medium used for panel D was collected from four experimental replicates utilizing two different HAE donors with one biological replicate from each donor. (E) Densitometry across conditions reported for panel D. Data represent MUC1-ED relative to actin for each sample, normalized to Mock/24 h. Results in panels B and E show data from three and four experimental replicates, utilizing three and four different PMD macrophage donors, with one biological replicate from each donor.

Beyond epithelial cells, MUC1 is known to be expressed by cells of the hematopoietic lineage (38–40), including macrophages, and this expression can modulate their phagocytic activity (35). As macrophages play an important role during IAV infection (41, 42) and because we observed elevated MUC1 protein during IAV infection and after IFN treatment across HAE component cell types, we next determined the impact of host- and virus-derived factors likely present in epithelial tissue during IAV infection on MUC1 expression in PMD macrophages. Following differentiation with granulocyte-macrophage colony-stimulating factor (GM-CSF; to better achieve alveolar-like macrophages) (43–45), we stimulated PMD macrophages with poly(I-C) (a viral double-stranded RNA mimetic), inflammatory cytokine TNF-α, type I interferon (IFN-β), or type III interferon (IFN-λ3) and assessed MUC1 expression in cell lysates by Western blotting. Although not as robust as the IFN-γ (type II IFN) and lipopolysaccharide (LPS) combination treatment (35), both poly(I-C) and IFN-β resulted in a strong upregulation of MUC1 protein, while IFN-λ3 and TNF-α induced detectable, albeit weak, upregulation (Fig. 5A and B).

To further assess whether MUC1 upregulation was mediated by soluble factors produced in the context of infection, we infected HAE with 5 × 10^4^ PFU (approximate MOI of 1) of A/Udorn/307/72, transferred the virus-free basolateral medium collected at 48 hpi (46) to naive PMD macrophages, and then assayed MUC1 protein expression in macrophage culture lysates 24 and 48 h later (Fig. 5C). While MUC1 protein was elevated by unspent and mock-conditioned medium, these levels were markedly increased in cultures receiving IAV-conditioned supernatant at both 24 and 48 h (Fig. 5D and E). These data indicate that IAV infection of HAE leads to the secretion of soluble factors that have the potential to increase MUC1 levels on multiple cell types during infection *in vivo*.

### Generation of HAE cultures lacking MUC1.

**FIG 6:**
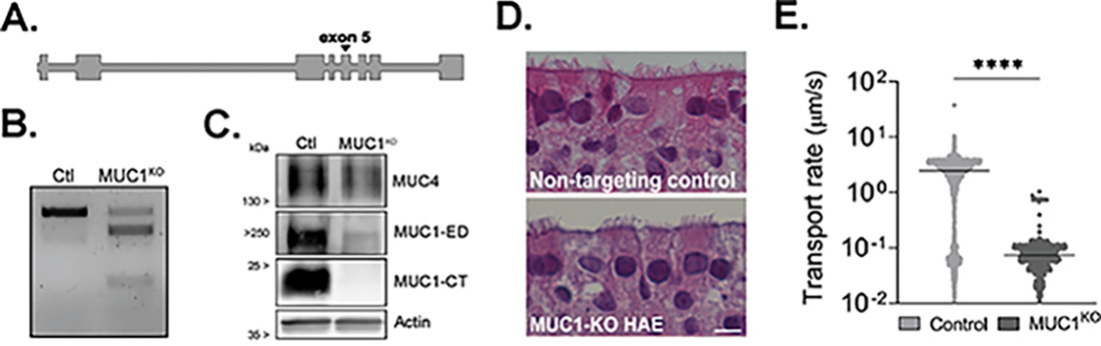
Establishment and characterization of immortalized HAE depleted of MUC1. Immortalized airway epithelial BCi-NS1.1 cells were transduced with CRISPR/Cas9 and sgRNA targeting (A) MUC1 exon 5 for protein depletion (MUC1^KO^) or without (control [Ctl]) the predicted targeting site. (B) Genomic DNA was extracted and used in a T7 endonuclease I cleavage assay demonstrating editing at the target site. (C) After differentiation, total HAE lysate was collected, separated by PAGE, and blotted for nontargeted tethered mucin MUC4 (extracellular domain), MUC1-ED, MUC1-CT, and actin. (D) Representative histological sections of paraffin-embedded cultures show normal ciliated epithelium. H&E counterstain. Bar = 20 μm. (E) Fluorescent microparticles were applied apically to indicated cultures to determine mucociliary transport rate. MCC between culture types was analyzed by the Mann-Whitney U test (****, *P* < 0.0001).

Given the ability of IAV to bind MUC1 during infection, and our observed changes in MUC1 protein dynamics in both HAE and PMD macrophages as a consequence of IAV infection, we next sought to determine the impact of MUC1 on IAV replication. We utilized CRISPR/Cas9-mediated genome editing to achieve well-differentiated HAE cultures that had genetically knocked-out (KO) MUC1. To do so, we cloned a single guide RNA (sgRNA) targeting MUC1 (exon 5) (Fig. 6A), or no known sequence (nontargeting control), into a green fluorescent protein (GFP)-expressing lentiviral vector that also encodes the Cas9 nuclease, transduced immortalized human airway epithelial cells (BCi-NS1.1 [47]), and sorted for GFP-positive cells prior to differentiation. Our data demonstrate on-target editing (Fig. 6B) and subsequent analysis using the Inference of CRISPR Edits (ICE) algorithm (48) revealed that 87% of alleles were edited in differentiated cultures, which correlated with a reduction in total MUC1 protein (Fig. 6C). We observed a lack of overt histopathology in differentiated cultures (Fig. 6D); however, compared with that in control cultures, MCC was significantly reduced in MUC1-depleted cultures (Fig. 6E). Nonetheless, overall, MUC1 was not critical for HAE differentiation or survival, allowing mechanistic dissection of its role in HAE.

### IAV challenge in HAE lacking MUC1 reveals altered infection dynamics.

**FIG 7:**
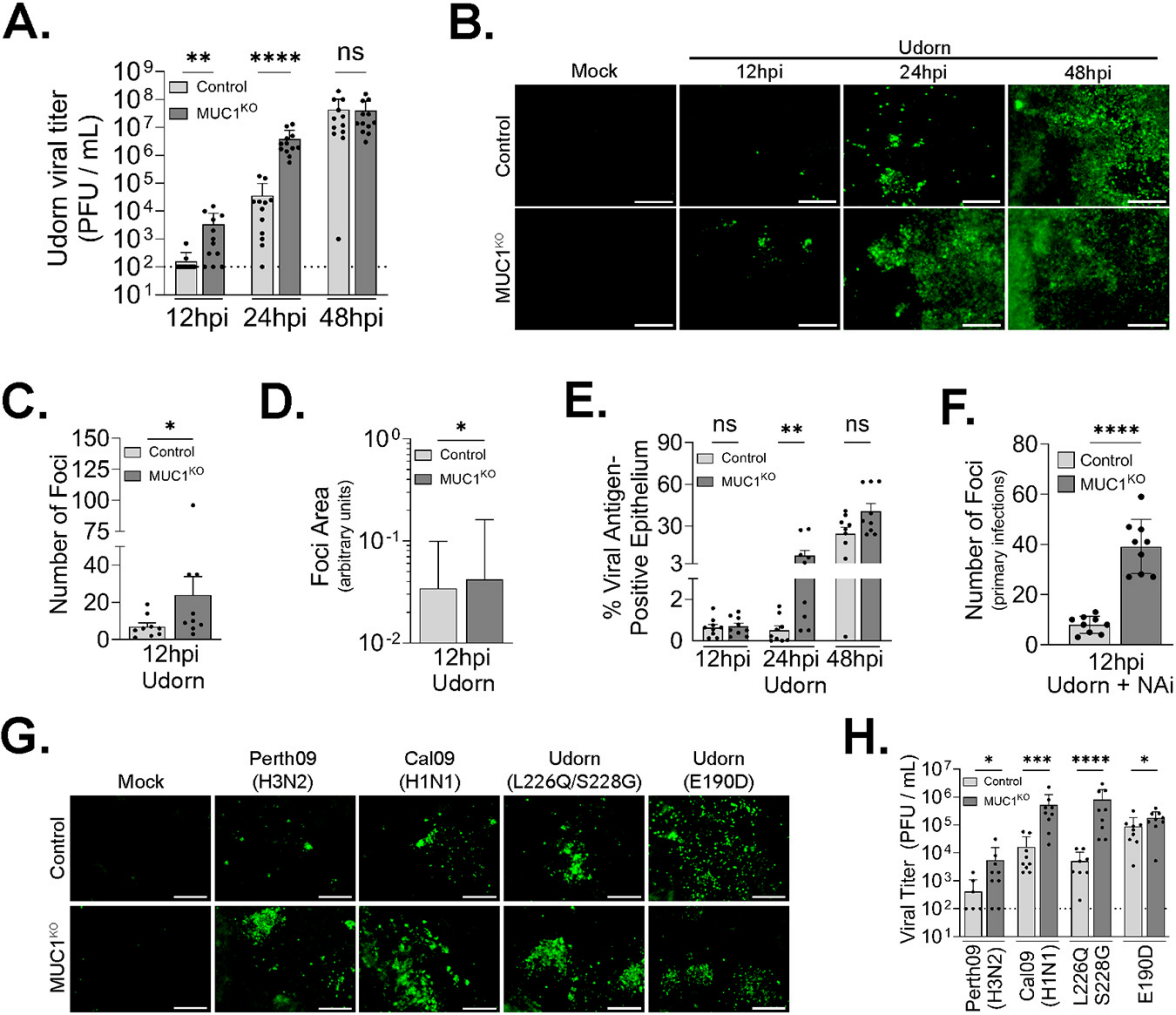
Infection in HAE lacking MUC1 with multiple IAV strains reveals enhanced viral spread. Well-differentiated control or MUC1^KO^ HAE cultures were infected with A/Udorn/307/72 at a low multiplicity of infection (5 × 10^2^ PFU; approximate MOI of 0.01). (A to E) At the indicated time points, cultures were washed apically with PBS for (A) viral titer determination, subsequently fixed, and (B) stained *en face* for viral nucleoprotein antigen. Viral antigen immunofluorescence signal at predetermined fields of view from HAE was analyzed for the (C) total number of fluorescent foci per individual HAE culture and (D) signal area of contiguous viral antigen (i.e., adjacent infected cells) by culture type. (E) The total viral antigen signal area per culture reported by collection time point. (F) HAE cultures were inoculated with A/Udorn/307/72 (5 × 10^3^ PFU, approximate MOI of 0.1) for 15 min prior to inoculum removal and addition of zanamivir (NAi) to both apical and basolateral compartments to prevent spread. Viral replication in initially infected cells (primary infections) was allowed to proceed for 12 hpi prior to fixation and focus quantification as for panel C. (G and H) Cultures were infected as for panels A to E with A/Perth/16/09 (H3N2), A/California/04/09(H1N1), A/Udorn/307/72(H3N2) with HA-L226Q/S228G, and A/Udorn/307/72(H3N2) with HA-E190D. At 24 hpi, the apical surface was washed with PBS to determine viral titer (H) and fixed to be stained for viral NP antigen (G). Data in panels C to E are from four experimental replicates; data in panel F are from three experimental replicates. Image analysis was performed using FIJI, and all results were analyzed by the Mann-Whitney U test. Data in panels A and H are from four and three experimental replicates, respectively. All data are significant where indicated (*, *P* < 0.05; **, *P* < 0.01; ***, *P* < 0.001; ****, *P* < 0.0001; ns = not significant). Bars = 100 μm.

To determine how MUC1 depletion would impact IAV infection dynamics, we inoculated both MUC1 KO and control HAE cultures with 500 PFU (approximate MOI of 0.01) A/Udorn/307/72 to allow multiple rounds of infection and monitored both viral growth kinetics as well as spread throughout the culture by *en face* staining for viral antigen. Viral titers were significantly higher in MUC1 KO cultures than control cultures at both 12 and 24 hpi; however, this difference was lost by 48 hpi (Fig. 7A). These data were consistent with immunostaining results, which revealed a limited number of viral antigen-positive cells in control cultures at 12 hpi, while all MUC1 KO cultures had resolvable foci indicative of multicycle replication by this time point (Fig. 7B). To assess whether IAV was better able to initiate successful infection of MUC1 KO cultures, we tabulated the number of viral antigen-positive foci on predetermined regions of infected cultures 12 hpi. MUC1 KO cultures had significantly more resolvable foci than control HAE cultures (Fig. 7C). We further expanded this analysis to assess the area of each identified focus and found that IAV foci were larger in MUC1 KO cultures (Fig. 7D). In line with these observations, MUC1 KO cultures also had a significantly greater percentage of viral antigen-positive epithelium at 24 hpi (Fig. 7E). By 48 hpi, both sets of cultures were extensively infected (Fig. 7B and E) and the integrity of the apical layer was severely compromised, with many regions entirely absent, indicating exhaustive infection in the culture and cytopathic effects (Fig. S5).

10.1128/mbio.01055-22.5 FIG S5 Relative cytotoxicity increases substantially at later time points during IAV infection in HAE. Relative cytotoxicity in MUC1-depleted and control HAE following IAV infection determined by quantification of lactate dehydrogenase levels in apical washes at indicated time points. Data are from three experimental replicates and were analyzed by the Mann-Whitney U test compared to control cultures of the same time point (ns, not significant; ****, *P* < 0.0001). Download FIG S5, TIF file, 2.8 MB. Copyright © 2022 Iverson et al. 2022 Iverson et al. https://creativecommons.org/licenses/by/4.0/ This content is distributed under the terms of the Creative Commons Attribution 4.0 International license.

To directly assess whether MUC1 impedes initial uptake of IAV in HAE, we inoculated control and MUC1 KO HAE cultures with 5,000 PFU of A/Udorn/307/72 (approximate MOI of 0.1) for 15 min. We then removed the inoculum and allowed replication to proceed up to 12 hpi in the presence of the neuraminidase inhibitor (NAi) zanamivir. These conditions were established to facilitate accumulation of viral antigen in initially infected cells but prevent further spread. Thus, we are able to assess the success rate of entry during this very short period. We then determined the frequency of successful infection events in the presence or absence of MUC1 by *en face* staining for viral nucleoprotein (NP) and quantifying NP^+^ foci in predetermined fields of view, as in Fig. 7C. Compared to control cultures, MUC1 KO cultures showed significantly more NP^+^ foci (Fig. 7F), supporting the hypothesis that MUC1 delays the productive uptake of IAV.

As the SA-binding capability of IAV is critical in mediating its endocytic uptake (27), and as we previously explored only the well-characterized lab strain A/Udorn/307/72, we sought to address whether MUC1’s anti-IAV functionality extends to more recent clinical isolates and A/Udorn/307/72 with altered SA-binding preferences. To address this question, we selected two viruses representing an H3N2 strain (A/Perth/16/09) and an H1N1 strain (A/California/04/09) circulating in humans in 2009. Additionally, we created two sets of mutations in the background of A/Udorn/307/72 (capable of binding to both α2-3- and α2-6-linked SA) which lead to enhanced recognition of either α2-3- (HA: L226Q/S228G) or α2-6-linked (HA: E190D) SA (49, 50). MUC1 KO and control HAE were infected as before, and viral titers in the apical compartment as well as the frequency of infected cells were assessed at 24 hpi. As we observed for A/Udorn/307/72 (Fig. 7B), all viruses displayed enhanced spread in MUC1 KO cultures compared to control cultures (Fig. 7G). Furthermore, in congruence with our earlier results, all viruses replicated to higher infectious titers in MUC1 KO cultures (Fig. 7H), although the magnitude of this difference varied between viruses. Together, these results indicate that under our experimental conditions, MUC1 is not required for initial attachment in HAE and, moreover, that its loss leads to enhanced viral replication and spread, particularly at early time points (Fig. 7A and H).

## DISCUSSION

It has been demonstrated that MUC1 plays an important, pathogen-specific, and potentially multifaceted role during respiratory infection (8, 14, 21, 30, 35). MUC1 is an abundant constituent of the PCL, where its extracellular domain contributes to airway surface hydration and its cytoplasmic domain has been shown to influence a variety of cellular signaling pathways that modulate the immune response (22, 51), cell survival (52, 53), and cancer progression (54). Additionally, MUC1 expression and phosphorylation state depend on external inflammatory stimuli (23, 24). Based on our previous work (33) and that of others (30), it is clear that MUC1 plays an important role during IAV infection. However, the nature of this role is poorly understood, and prior research was done in cell culture systems lacking a well-developed glycocalyx or in mice, where mucin orthologs exhibit incomplete homology with their human counterparts. Thus, we sought to explore MUC1-IAV interaction, dynamics of expression, and overall impact on IAV infection in a physiologically relevant *in vitro* model of human airway epithelium.

Our results support a direct interaction between IAV and endogenous MUC1 during infection in HAE, extending previous findings that demonstrated colocalization of IAV with MUC1 on the surface of A549 cells (30). Notably, MUC1-ED, the large extracellular domain of MUC1, is capable of dissociating from MUC1-CT through the autocatalytic SEA module in response to external stimuli (10, 55), and it has been suggested that this cleavage domain facilitates release of MUC1-ED upon interaction with IAV in the airway lumen (30). However, we did not detect an increase in soluble MUC1-ED levels after IAV infection in either HAE or A549 cells, suggesting that IAV binding to MUC1-ED does not induce its shedding in systems with endogenous expression with or without a dense glycocalyx.

Surprisingly, we found that type I and type III IFN can upregulate cell-associated MUC1 protein in HAE despite no significant increases in MUC1 mRNA levels. These data hint at the possibility that some MUC1 expression is regulated through a posttranscriptional mechanism under these conditions. IAV upregulation of MUC1 protein in HAE was not exclusively dependent on IFN signaling, indicating that multiple soluble factors produced during infection may contribute to elevated MUC1 expression. At least part of this increased expression was due to MUC1 upregulation at the apical surface, though broad expression of MUC1 across all HAE component cell types after IAV infection and after IFN stimulation further indicates that MUC1 expression is nearly ubiquitous across the epithelium. While upregulation at the apical surface likely contributes to barrier function, expression here and in other cell types (e.g., basal cells) may play alternative roles, potentially suppressing inflammation (51) and/or priming for epithelial repair in response to damage (9, 54, 56).

As macrophages play a key role during IAV infection (41, 42) and previous work demonstrated that macrophages can express MUC1 in response to type II IFN (35), we explored whether IFN produced during IAV infection (36) could induce MUC1 in PMD macrophages. We show here that, in addition to HAE, PMD macrophages upregulate MUC1 following type I and type III IFN stimulation. While literature on the human monocyte response to type III IFN is conflicting (57), human monocyte-derived macrophages and *ex vivo* human macrophages are capable of responding to type III interferon (57–59), which is consistent with our observations across multiple donors. Moreover, these PMD macrophages upregulate MUC1 in response to soluble factors produced by infected HAE. These results suggest that sites of infected epithelium might induce MUC1 expression in local macrophages as well as potentially other immune effector cells that have been shown to at least conditionally express MUC1 (38–40). Interestingly, the banding pattern of MUC1-ED as expressed in PMD macrophages suggests an altered glycosylation state. As the expression (35) and glycosylation state (60) of MUC1 can both independently influence uptake of foreign material in different cellular contexts, further investigation should be undertaken to explore cell-specific impacts of MUC1 expression during IAV infection.

We have also established a MUC1-depleted HAE system through CRISPR/Cas9 technology. Others have established similar workflows (61, 62) which offer the powerful ability to genetically manipulate otherwise intractable primary human tissue. Our characterization of these immortalized KO cultures reveals robust protein depletion as well as no gross morphological pathology. Additional functional characterization, however, revealed that MUC1-depleted cultures displayed markedly lower MCC than nontargeting control cultures. As the PCL contributes to airway hydration and therefore proper secreted mucus mobility (1–3), it follows that MUC1 depletion could negatively affect this capability. It is also possible that loss of MUC1 alters other factors which impact MCC, such as baseline secreted mucin expression, which were not measured in this study. Future studies on air surface liquid characteristics such as PCL density and/or height, combined with other mucus steady-state kinetics (e.g., secreted mucin expression), will better delineate the contribution MUC1 and other tethered mucins make toward overall mucociliary function. The HAE system we utilized here is one of several *in vitro* models that offer the ability to probe the mucosal interface, which has been difficult to study in normal two-dimensional (2D) tissue culture systems (32).

Consistent with other findings (30), we found that IAV can interact with MUC1; however, in our HAE system depleted of MUC1, we observed that IAV growth kinetics are increased over control cultures, particularly at 12 and 24 hpi. Importantly, recent work with Muc1 knockout mice has similarly shown that the loss of Muc1 enhances the rate of IAV replication, though this did not impact the cumulative viral load (63). In our HAE system, MUC1-depleted cultures had detectable viral titers at the earliest time point of 12 hpi, whereas the majority of control cultures were below the limit of detection. Additionally, not only was the number of foci detectable by *en face* immunofluorescence significantly higher, but there was also clear evidence of multicycle replication visible as early as 12 hpi (the earliest time point investigated) in MUC1 KO cultures compared to control cultures. Since IAV can produce new virions as early as 6 h (64), this implies that there is a significant delay in both the timing and success rate of productive infection initiation in control cultures relative to MUC1-depleted cultures. Indeed, the significant difference in successful infection events after just 15 min of inoculation time suggests that a more rapid initial uptake of viral particles contributes to the enhanced replication and spread of IAV in MUC1-depleted cultures. This supports a model whereby MUC1 provides significant contribution in inhibiting and delaying IAV access to productive endocytic events and is consistent with previous reports of the PCL restricting viral access to the apical membrane of epithelial cells (17) as well as synthetic models of tethered mucins which inhibited and delayed IAV-mediated fusogenic events (65). In the native airway environment, tethered mucins of the PCL affording cells such a dramatic delay in uptake could severely limit the ability of IAV to establish an infection before being cleared from the lung. We note that in the HAE culture system, there is no true clearance resulting from MCC, as the secreted mucus layer and its components are transported over the same regions of the culture endlessly.

One current model for IAV uptake suggests that virions rely on multivalent interactions with sialylated host proteins and glycolipids to deform local membrane orientation and subsequently trigger endosomal uptake (66, 67). While neuraminidase activity is normally thought of as a mechanism to avoid virion aggregation and inhibition by secreted mucins (68), recent work has additionally highlighted its importance at this early entry step at or near the host cell membrane (69, 70). In this model, tethered mucins support virion clearance through air-surface liquid hydration and MCC (1, 2), but also, as large constituents of the PCL, they sterically block and, when sialylated, compete with productive virion attachment to membrane-adjacent sialylated attachment sites (1, 2).

Our results obtained with wild-type Udorn (which binds both α2-3- and α2-6-linked SA) and the mutant Udorn L226Q/S228G and E190D viruses possessing altered SA-binding profiles indicate that MUC1 can inhibit IAV replication regardless of this receptor preference. Notably, the α2-6-linked SA-binding Udorn mutant (E190D) displayed a much smaller difference in replication between MUC1 KO and control cultures relative to both the α2-3-linked SA-binding mutant (L226Q/S228G) and wild-type Udorn. Viruses with a preference for α2-3-linked SA might be more inhibited by MUC1 relative to those with a mixed or α2-6-linked dominant binding profile. However, compared to wild-type Udorn, both SA-binding mutants yielded lower titers at 24 hpi in MUC1 KO cells. Previous work has shown that HA receptor binding preference fitness is also significantly reliant on epistatic balance even between residues outside the receptor binding domain (71), which can confound conclusions about MUC1’s influence on these mutants. Additionally, MUC1 significantly inhibited the replication of both A/Perth/16/09 and A/Cal/04/09 clinical isolates. Seasonal H3N2 and H1N1 viruses have converged in their human receptor preferences for α2-6-linked SA-containing glycoconjugates (72), though more modern drifted H3N2 variants might have continued to diverge in this regard (73). Both of these clinical isolates display a wider degree of enhanced replication in MUC1 KO relative to control cultures (1.1- and 1.5-log-scale difference for A/Perth/16/09 and A/California/04/09, respectively) compared to the α2-6-linked SA-binding Udorn mutant (0.3 log). It is also possible that preference for the carbohydrate core in addition to the terminal sialylated moiety further influences the inhibitory function of MUC1. Nonetheless, work on artificial tethered mucin analogs has shown that both sialylated and unsialylated artificial tethered mucins can inhibit productive interactions with gangliosides and delay IAV fusion events, respectively (65). Together, these results suggest that, regardless of receptor binding, MUC1 can significantly inhibit IAV spread in HAE.

Our results are also consistent with the emerging role of MUC1 in response to inflammatory stimuli, and we expand on known inflammatory triggers for its expression both in HAE and in PMD macrophages. Indeed, the surprising finding that MUC1 is upregulated beyond the apical layer supports a broader dynamic role during infection at the epithelial surface. Specifically, our data support the model proposed by Kato et al. (51) whereby pathogenic insult leads to general inflammation that subsequently upregulates MUC1 expression and recruits immune cells (63). This would immediately protect local epithelial cells by acting as a barrier, but further accumulation would help resolve potentially harmful inflammation and simultaneously prime cells for survival and ultimately proliferation to repair local tissue damage following infection.

Additionally, our results demonstrate that MUC1 significantly reduces IAV replication by acting early in infection, consistent with its canonical role as a barrier protecting the airway epithelium. However, instead of the model that suggests that MUC1 is acting as a soluble decoy receptor that is dynamically shed in response to viral interaction, our work indicates that MUC1 acts as a general barrier to productive endocytic uptake. As we investigated only the earliest steps in IAV infection of HAE, future studies should interrogate how viral-mediated upregulation of MUC1 may impact subsequent spread and immune response to an established infection.

## MATERIALS AND METHODS

### Human airway epithelial cultures.

Human airway tracheobronchial epithelial cells isolated from airway specimens from donors without underlying lung disease were provided by Lonza, Inc. The immortalized HAE line BCi-NS1.1 was kindly provided by Matthew Walters and Ronald Crystal (Weill Cornell Medical College) (47). Both primary cells derived from single patient sources and BCi-NS1.1 airway epithelial cells were first expanded on plastic in Pneumacult-Ex or Pneumacult-Ex Plus medium (no. 05008 or 05040, StemCell Technologies). Airway cells were then seeded (3.3 × 10^4^ cells/well) on rat tail collagen type 1-coated permeable Transwell membrane supports (6.5 mm; no. 3470, Corning, Inc.) and differentiated in Pneumacult-ALI medium (no. 05001, StemCell Technologies) or custom ALI media (Spirovation, UNC Marsico Lung Institute) with provision of an air-liquid interface for approximately 6 weeks to form polarized cultures that resemble *in vivo* pseudostratified mucociliary epithelium. All cell cultures were maintained at 37°C with 5% CO_2_. All experiments utilized at least two different donors, and the data in the figures are indicative of unique biological replicates.

### Primary human macrophage cultures.

Peripheral blood was collected from healthy volunteers, and mononuclear cells were separated by Ficoll‐Hypaque density gradient centrifugation. Monocytes were isolated by adherence to plastic and then cultured for 1 week in X-VIVO 15 serum-free medium (no. BE02-060Q, Lonza, Inc.) with 20 ng/mL recombinant human GM-CSF (no. 300-03, Peprotech). Medium containing GM-CSF was replenished 4 days after initial culture. Prior to stimulation (see “Cell culture and cell culture treatments” below), GM-CSF-containing medium was removed and replaced with X-VIVO 15 medium supplemented with 5% fetal bovine serum (FBS; no. 25-514H, Genclone). For HAE medium stimulations, ALI medium (comprising an additional 25% volume) was added to X-VIVO 15 medium supplemented with 5% FBS at 24 and 48 h prior to lysate collection. All studies on human monocyte‐derived macrophages were approved by the University of Maryland Institutional Review Board, and formal written consent was obtained where necessary.

### MUC1 immunoprecipitation.

MUC1 antibody (235 μg, clone B27.29, a gift from Fujirebio Diagnostics Inc.) was conjugated to aldehyde/sulfate latex beads (six drops; no. A37384, Invitrogen) through constant rotation at room temperature for 2 h. Following incubation with anti-MUC1 antibody and subsequent washing with phosphate-buffered saline (PBS), beads were incubated with 1 M glycine and 0.5% bovine serum albumin (BSA; no. BP1600100, Fisher Scientific) at room temperature for 30 min without agitation to coat any remaining exposed area and prevent nonspecific binding of protein during immunoprecipitation. HAE apical secretions (100 μL of PBS culture wash) were pretreated with Triton-X (final concentration = 0.1%) before mixing with anti-MUC1-conjugated beads. Following overnight incubation at 4°C with constant inversion, the beads were washed twice with PBS and then resuspended in Tris-glycine SDS-PAGE sample buffer (no. LC2676, Invitrogen) and sample reducing agent (no. NP0009, Invitrogen) with total input serving as a control. Samples were vortexed and heated to 95°C for 5 min before loading into a 4 to 20% Tris-glycine SDS-PAGE gel in triplicate (no. XP04202BOX, Invitrogen) for electrophoresis. For blotting, membranes were blocked with 5% milk and Tris-buffered saline (167.8 mM Tris-HCl; 32.0 mM Trizma base, 1.5 M NaCl) with 0.1% Tween 20 (TBS-T) before incubation with one of the following, in parallel: anti-MUC1 antibody (1:5,000; clone B27.29; a gift from Fujirebio Diagnostics, Inc.), recombinant H3-Fc (1 μg; a gift from Ian Jones and Silvia Loureiro), or anti-MUC16 antibody (1:5,000; clone X325; no. ab10033, Abcam). Recombinant hemagglutinin proteins were generated by infection of insect cells with a recombinant baculovirus expressing the protein as previously described (74). Blots were then probed with the secondary antibody corresponding to the primary probe (anti-mouse IgGκ-HRP [1:10,000; no. sc-516102, Santa Cruz] or anti-human IgG Fc-HRP [1:10,000; no. A18829, Invitrogen]) prior to reaction with SuperSignal West Dura substrate (no. 34075, Thermo Scientific) and imaging on the iBright 1500 (Thermo Fisher) machine.

### ELISA.

Soluble MUC1 was quantified by ELISA (no. EHMUC1, Invitrogen) according to the manufacturer’s protocol. To collect HAE samples prior to analysis, 50 μL PBS was applied to the apical chamber and incubated for 30 min at 37°C. Prior to experimentation in A549 adenocarcinoma human alveolar basal epithelial cells, regular growth medium (high-glucose Dulbecco’s modified Eagle medium [DMEM] [no. 11-965-092, Gibco] supplemented with 10% fetal bovine serum) was replaced with serum-free DMEM (no. 11-965-092, Gibco). HAE culture washes and A549 culture supernatants were stored at −80°C prior to analysis. Total soluble MUC1 was calculated based on concentration determined by ELISA and total volume collected.

The MUC1 ELISA (no. EHMUC1, Invitrogen) was also adapted to detect MUC1 with biotinylated influenza virus (see “Influenza viruses”). Here, the standard curve and HAE lysate (25 μg) diluted in diluent B were incubated in the wells at room temperature for 2.5 h without agitation. Afterwards, the plate was transferred to ice with sample removal and washing with prechilled wash buffer. All subsequent washing steps were carried out with prechilled washing buffer. Prechilled, biotin-conjugated MUC1 detection antibody, biotinylated virus (Udorn^biotin^, diluted 1:10 in diluent B), and biotinylated virus mixed with neutralizing antibody (1:1,250, anti-Hong Kong/68 goat antiserum [no. NR-3118, BEI Resources]) were added to the plate for 1 h on ice. Biotin-conjugated MUC1 detection antibody and biotinylated virus were removed, the plate was washed, and prechilled streptavidin-HRP was added to the plate for 45 min on ice. After removal of the streptavidin-HRP and washing, the plate was taken off ice, allowed to equilibrate to room temperature for 15 min, and UV sterilized. Finally, TMB (3,3′,5,5′-tetramethylbenzidine) substrate was added to the plate, and the reaction mixture was incubated for 30 min in the dark at room temperature prior to the addition of stop solution and sample reading.

### Influenza viruses.

The reverse genetics systems for A/Puerto Rico/8/1934 (H1N1; PR8), A/Udorn/307/72 (H3N2; Udorn), and A/Perth/16/09 (Perth09) (75) were generous gifts from Adolfo Garcia-Sastre, Robert Lamb, and Jesse Bloom, respectively. Reverse genetics plasmids for viruses utilizing the pDZ backbone (i.e., PR8 and Perth09) were validated using the primer 5′ GTG TGT CCT GGG GTT GAC CA 3′. Reverse genetics helper plasmids for Udorn (pHW2000) and segment-specific plasmids (pHH21) were verified using the pPolI reverse primer 5′ ATG GTG GCG TTT TTG GGG ACA 3′. Live A/California/04/09 (Cal09) virus was purchased from BEI Resources (NR-13658), total RNA was extracted (no. 74104, Qiagen) from clarified viral supernatant per the manufacturer’s instructions and processed into cDNA (no. 18080092, Invitrogen); and each segment’s identity was validated using segment-specific forward and reverse primers as previously described (76).

To produce Udorn viruses with altered SA binding, HA mutations at positions H3 E206D (E190D; H1 numbering) and H3 L242Q/S244G (L226Q/S228G; H1 numbering) were introduced into the A/Udorn/307/72 reverse genetics system. Specifically, E206D was achieved through digestion of the segment 4 plasmid with HindII and XbaI to insert a gBlock (segment 4 nucleotides 332 to 757) containing the GAA to GAU transversion which is predicted to be codon optimized for both canines and humans. For both L242Q and S244G, the segment 4 plasmid was digested with XbaI and XhoI to insert a gBlock (segment 4 nucleotides 712 to 1327). Specifically, L242Q was achieved through CTG-to-CAA double mutation rather than the single and human codon-preferred CAG transversion, as a greater barrier to reversion mutation. S244G was achieved through AGT-to-GGA double mutation to avoid GC/CG dinucleotide bias and avoid codon bias in both canine and human hosts. For viruses derived directly from reverse genetics systems, infectious virus stocks were produced by plasmid transfection in 293T cells and subsequent coculture with Madin-Darby canine kidney (MDCK) cells (77). Notably, Udorn E190D and L226Q/S228G mutant virus stocks were sequenced after rescue to ensure retention of the introduced nucleotide changes. Afterward, all viruses were amplified by passage in MDCK cells as described below.

To amplify influenza virus stocks, confluent MDCK monolayers were inoculated (MOI = 0.01 or 0.001) in the presence of 1.5 μg/mL TPCK (tosylsulfonyl phenylalanyl chloromethyl ketone) trypsin-supplemented, serum-free, high-glucose DMEM, and infection was allowed to proceed for 72 h or until at most 25% of cells remained adherent. Sodium bicarbonate (NaHCO_3_) was added as needed to maintain neutral pH, and the supernatant was clarified with a 1,000 × *g* spin at 4°C for 15 min. Clarified supernatant was concentrated and purified at 4°C through 20% sucrose (solubilized in NTE buffer; 100 mM NaCl, 10 mM Tris [pH 7.4], 1 mM EDTA) layered on a 50% sucrose NTE cushion by centrifugation at 100,000 × *g* (25,000 rpm; SW-41Ti; calculated relative to the bottom of the bucket) for 2 h. Virus at the interface was collected, mixed thoroughly by pipetting and vortexing, and then aliquoted for storage at −80°C. Once frozen, an aliquot was used to determine virus titer by plaque assay on MDCK cells. Briefly, confluent monolayers of MDCK cells in 12-well plates were washed with PBS prior to addition of 100 μL of viral inoculum diluted in serum free DMEM. This was incubated with periodic agitation for 1 h at 37°C before being aspirated and replaced with 0.8% molten agar in DMEM/F-12 (no. 12500062, Gibco) and 1.5 μg/mL TPCK trypsin. After agar solidification, plates were inverted and incubated at 37°C for 72 h prior to plaque counting.

For biotinylation of influenza virus, sucrose purified A/Udorn/307/72 (4.7 × 10^7^ PFU/mL) was dialyzed against 0.1 M carbonate (NaHCO_3_) and 100 mM NaCl reaction buffer. EZ-Link sulfo-NHS-SS-biotin (no. 21331, Thermo Scientific) was freshly solubilized in dimethyl sulfoxide (DMSO) before addition to the virus in the reaction buffer at a final concentration of 1 μM. The virus-biotin mixture was incubated on ice for 20 min with gentle shaking before addition of more biotin at the same final concentration, minimizing added volume. After an additional 20-min incubation, the reaction was quenched with chilled 50 mM glycine for 10 min. Labeled virus was then dialyzed overnight against PBS before being aliquoted and stored at −80°C. Biotin labeling was confirmed by Western blotting (see “Western blotting” below), and infectivity was subsequently analyzed by plaque assay. To confirm that labeled virus retained infectivity and tropism for both ciliated and nonciliated cells on HAE cultures, 25 μL of labeled virus was allowed to adsorb for 2 h prior to inoculum removal. The infection was allowed to proceed for 24 h prior to culture fixation and staining for both viral antigen and ciliated cell markers.

### Influenza virus infection in HAE.

For infection in unmodified HAE, cultures were washed with PBS for 15 min at 37°C to remove apical secretions and supplied with fresh basolateral medium prior to inoculation with sucrose-purified virus diluted in PBS to a final volume of 50 μL. Inoculum was applied to the apical surface of HAE for 2 h at 37°C. Following incubation, viral inocula were removed, and cultures were washed once with PBS for 10 min to remove unbound virus.

To better mimic natural infection for kinetic analysis in CRISPR/Cas9-modified, BCi-NS1.1-derived HAE, cultures were washed with PBS for 30 min at 37°C and then incubated for 7 days to allow recovery of the secreted mucus layer prior to inoculation. In these experiments, sucrose-purified viruses were diluted to 500 PFU (approximate MOI of 0.01) in 10 μL PBS, and inocula were not removed. For all experiments, progeny virus was harvested at indicated times by performing apical washes with 50 μL of PBS for 30 min at 37°C and stored at −80°C prior to analysis. To measure cytotoxicity, lactate dehydrogenase (LDH) in apical washes was measured with CytoTox 96 (no. G1780, Promega) according to the manufacturer's instructions.

To assess influenza virus entry efficiency, CRISPR/Cas9-modified, BCi-NS1.1-derived HAE were left unwashed for 7 days as described above prior to the application of 5,000 PFU (approximate MOI of 0.1) of A/Udorn/307/72 to the apical surface in a 30-μL volume. After a 15-min incubation, viral inocula were removed and apical and basolateral compartments were replaced with PBS and standard ALI medium, respectively, supplemented with a 1,250 nM concentration of the neuraminidase inhibitor zanamivir (no. SML0492, Sigma-Aldrich). The cultures were then returned for incubation at 37°C until 12 hpi, when they were fixed and processed for viral antigen staining *en face* as described below.

### qRT-PCR.

RNA was extracted using an RNeasy minikit (no. 74106, Qiagen) according to the manufacturer's instructions. cDNA was prepared separately with SuperScript III (no. 18080044, Invitrogen) per the manufacturer’s random-hexamer protocol. For quantitative PCR (qPCR), reactions were carried out using LightCycler 480 SYBR green I master mix (no. 04-707-516-001, Roche) and a LightCycler 480 II instrument (Roche) at the manufacturer’s recommended settings. Primer sequences, if available, are listed in Table 1.

**TABLE 1:** Primers

Gene target	Primer	
	Forward (5′–3′)	Reverse (5′–3′)
MUC1	Qiagen, proprietary (QT00015379)	Qiagen, proprietary (QT00015379)
HPRT1	Qiagen, proprietary (QT00059066)	Qiagen, proprietary (QT00059066)
MX1 (ENSG00000157601)	GTTTCCGAAGTGGACATCGCA	CTGCACAGGTTGTTCTCAGC
IL-8 (ENSG00000169429)	GAATGGGTTTGCTAGAATGTGATA	CAGACTAGGGTTGCCAGATTTAAC

### Western blotting.

Protein lysate was collected with radioimmunoprecipitation assay (RIPA) buffer (no. 10191-284, VWR Life Science) supplemented with 2× protease inhibitors (no. A32963, Thermo Scientific). Protein concentration was quantified by bicinchoninic acid (BCA) assay (no. 23225, Thermo Scientific), loaded equivalently in each lane (ranging from 4 to 20 μg between experiments), and run on a 4 to 20% Tris-glycine gel (no. XP04205BOX, Invitrogen) under reducing conditions. Western blot analysis of biotinylated virus was run under nonreducing conditions. Protein was transferred to a polyvinylidene difluoride (PVDF) membrane (no. 10600030, Cytiva) and blocked with 5% (wt/vol) fat-free milk protein in TBS-T at room temperature. Incubations with unconjugated primary antibody were done in the presence of blocking protein and TBS-T overnight at 4°C. Antibodies used were MUC1-CT (clone MH1 [CT2]; no. MA5-11202, Invitrogen; 1:5,000), MUC1-ED (clone B27.29; a gift from Fujirebio Diagnostics Inc.; 2.04 μg/mL), MUC4 (clone 1G8; no. sc-33654, Santa Cruz; 1:5,000), and MX1 (clone N2C2, no. GTX110256, GeneTex, 1:5,000). After washing in TBS-T, membranes were probed with secondary antibodies for 1 h at room temperature in blocking buffer. Specifically, anti-mouse IgGκ-HRP (no. sc-516102, Santa Cruz; 1:10,000), anti-Armenian hamster IgG-HRP (no. PA1-32045, Invitrogen; 1:10,000), and anti-rabbit-HRP (no. 32460, Invitrogen; 1:10,000) were used to image MUC1-ED and MUC4, MUC1-CT, and MX1, respectively. Actin was detected using an HRP-conjugated primary antibody (clone AC-15; no. A3854, Sigma-Aldrich; 1:35,000) for 1 h at room temperature in blocking buffer with rocking. Imaging was performed with chemiluminescent SuperSignal Dura or Femto reagent (no. 34075 or 34095, Thermo Scientific) on an iBright 1500 (Thermo Fisher). Densitometry for Fig. 3 and 5 was performed using ImageJ analysis of select band intensity (MUC1-CT or MUC1-ED where indicated) relative to same-sample actin band intensity. Within an experimental replicate, results for individual samples were then normalized to results for the samples indicated (represented by a normalization value of 1.0). MUC1-CT antibodies were used preferentially for Western blot densitometry in HAE due to the smaller size and lack of glycosylation on this part of the mucin. MUC1-ED antibodies were used for densitometry analysis of MUC1 in PMD macrophage experiments, as MUC1 expression is significantly lower than that of HAE. The MUC1-ED-directed antibody detects a repeated epitope in the VNTR region of MUC1 that enhances sensitivity.

### Cell culture and cell culture treatments.

MDCK cells were a generous gift from Wendy Barclay. They were maintained at 37°C and 5% CO_2_ in high-glucose DMEM (no. 11-965-092, Gibco) supplemented with 10% fetal bovine serum and passaged at 100% confluence with 0.25% trypsin-EDTA (no. 25200-072, Gibco). HEK293T and A549 cells were both purchased through ATCC (no. CRL-11268 and CCL-185). Both HEK293T and A549 cell lines were maintained at 37°C and 5% CO_2_ in high-glucose DMEM (no. 11-965-092, Gibco) supplemented with 10% fetal bovine serum and passaged at 80 to 90% confluence with 0.05% trypsin-EDTA (no. 25300-062, Gibco). All cell lines were routinely tested for the presence of mycoplasma (MycoStrip; no. rep-mys-50, InvivoGen). Unless otherwise specified, recombinant human IFN-β (1 nM; no. 11415-1, PBL Assay Science), IFN-λ3 (10 nM; no. 11730-1, PBL Assay Science), ruxolitinib (2 μM; no. S1378, SelleckChem), and DMSO (no. ATCC-4-X, ATCC) were applied to cell culture media or to both the apical and basolateral chambers of HAE cultures. TNF-α (20 ng/mL; no. 210-TA, R&D Systems) was applied apically to HAE cultures. For experiments with primary macrophage cultures, IFN-β (1 nM), IFN-λ3 (10 nM), TNF-α (20 ng/mL), low-molecular-weight poly(I-C) (10 μg/mL; k-picw, InvivoGen), and LPS (100 ng/mL E. coli K-12; k-eklps, InvivoGen) with IFN-γ (20 ng/mL; no. 285-IF-100/CF, R&D Systems) were used to supplement X-VIVO 15 medium and 5% fetal bovine serum at indicated time points prior to lysis.

### Immunohistochemistry (IHC) and immunofluorescence (IF) microscopy.

HAE cultures were fixed in 4% paraformaldehyde overnight prior to paraffin embedding and sectioning at either the Marsico Lung Institute Histology Core (Chapel Hill, NC) or the New York University Experimental Pathology Research Laboratory (New York, NY). Five-micrometer-thick sections on slides were deparaffinized with xylene and rehydrated through gradient ethanol washes into distilled water. For staining of MUC1-CT and parallel targets, heat antigen retrieval was performed as follows. Citrate buffer (2.94 g/L), pH 6.0, with 0.05% Tween 20 was heated to 98°C to boil deparaffinized slides for 15 min. After the slides were cooled and washed in water, they were blocked with 3% BSA in PBS supplemented with 1 mM CaCl_2_ and 1 mM MgCl_2_ (PBS++) for 10 min at room temperature. Primary antibodies were diluted in 1% BSA–PBS++ and incubated with the sample overnight at room temperature. Slides were then washed with PBS++ and secondary antibodies (also diluted in 1% BSA–PBS++) added for 1 h at room temperature. Slides were then stained with 4′,6-diamidino-2-phenylindole dihydrochloride (DAPI; no. D1306, Invitrogen) or Hoechst 33342 solution (no. H3570, Thermo Scientific) and washed a final time with PBS++, and coverslips were mounted with Vectashield antifade mounting solution (no. H-1000, Vector Laboratories).

Antibodies for IHC included acetylated alpha-tubulin (cilium marker, primary antibody; 1:2,000; clone 6-11B-1; ab24610, Abcam), anti-mouse IgG2b-Alexa Fluor 488 (1:250; no. A-21141, Invitrogen), MUC1-CT (primary antibody; 1:150; clone MH1 [CT2]; no. MA5-11202, Invitrogen), anti-Armenian hamster IgG-Alexa Fluor 647 (secondary antibody; 1:500; no. ab173004, Abcam), rH3-Fc probe (primary probe; a gift from Ian Jones and Silvia Loureiro; 1 μg), anti-human IgG Fc (secondary antibody; 1 μg; no. 31125, Invitrogen), anti-goat IgG-Alexa Fluor 488 (1:500; no. A-11055, Invitrogen), MUC1-ED (primary antibody; 1:4,600; clone B27.29; a gift from Fujirebio Diagnostics Inc.), MUC16 (primary antibody; 1:1,000; clone X325; no. ab10033, Abcam), MUC4 (primary antibody; 1:1,000; clone 1G8; no. sc-33654, Santa Cruz), and anti-mouse IgG-Alexa Fluor 488 (secondary antibody; 1:500; no. A-11001, Invitrogen) or anti-mouse IgG-Alexa Fluor 555 (secondary antibody; 1:250. no. A-31570, Invitrogen).

To prepare HAE cultures for *en face* IF staining, cultures were fixed in 4% paraformaldehyde for 20 min at room temperature, permeabilized with 2.5% Triton X-100, and blocked with 3% BSA in PBS++. The IF antibody staining procedure was the same as for IHC.

For IF, antibodies were as follows: acetylated alpha-tubulin (1:2,000; clone 6-11B-1; no. ab24610, Abcam), anti-mouse IgG2b-Alexa Fluor 488 (1:500; no. A-21141, Invitrogen), MUC1-CT (1:50; clone MH1 [CT2]; no. MA5-11202, Invitrogen), anti-Armenian hamster IgG-Alexa Fluor 647 (1:500; no. ab173004, Abcam), influenza virus NP (1:100; clones A1 and A3; no. MAB8251, Sigma-Aldrich), and anti-mouse IgG-Alexa Fluor 488 (no. A-11001, Invitrogen; 1:500). MUC1-CT was utilized to accommodate the use of other mouse antibodies in concurrent staining. Images were acquired with a Zeiss Axio Observer 3 inverted fluorescence microscope equipped with a Zeiss AxioCam 503 mono camera and Zen imaging software.

### Transmission electron microscopy.

For transmission electron microscopy detection of virus and MUC1, two protocols were used. In the first, HAE cultures were washed and 4.7 × 10^6^ PFU (approximate MOI of 94) of sucrose-purified A/Udorn/307/72 was allowed to adsorb for 1 h at 37°C, followed by transfer of the cultures to 4°C for all subsequent steps up to fixation (Fig. 1H). In the second, HAE cultures were washed and 5 × 10^5^ PFU (approximate MOI of 10) of dialyzed, sucrose-purified A/Udorn/307/72 was allowed to adsorb for 2 h at 4°C (Fig. 1F, G, and I). The virus inoculum was removed, and cultures were blocked with 10% (vol/vol) normal donkey serum for 1 h. Anti-MUC1-ED B27.29 (2.04 μg/mL) and anti-Hong Kong/68 goat antiserum (no. NR-3118, BEI Resources) were added in the presence of blocking serum overnight. Cultures were washed with PBS++ to remove primary antibodies before addition of 18-nm-gold-conjugated anti-mouse (1:10; no. 115-215-166, Jackson ImmunoResearch Laboratories, Inc.) and 6-nm-gold-conjugated anti-goat antibodies (1:20; no. 705-195-147, Jackson ImmunoResearch Laboratories, Inc.) in blocking solution for 1 h. Secondary antibodies were then removed and cultures were washed and subsequently fixed in 2% glutaraldehyde in 0.1 M cacodylate buffer for 1 h at room temperature. Following a further washing step in 0.1 M cacodylate buffer, a secondary fixation step using 1% OsO_4_ and 1% K_3_Fe(CN)_6_ in 0.1 M cacodylate buffer was performed for 1 h. A final wash of 0.1 M cacodylate buffer was performed before postfixation treatment with 2% uranyl acetate solution in distilled H_2_O for 1 h. Cultures were then dehydrated in increasing concentrations of ethanol. Finally, cultures were infiltrated with 100% propylene oxide and subsequently with increasing ratios of Spurr’s resin up to the final embedding step of 100% Spurr’s resin. Cultures were then imaged at 80 kV on the Hitachi HT7700 transmission electron microscope at the Laboratory for Biological Ultrastructure at the University of Maryland.

### Mucociliary clearance.

HAE mucus was allowed to accumulate for 1 week prior to the application of 5 μL of 2-μm red-fluorescent (Cy3) polystyrene microspheres (1:50, no. F8826, Invitrogen) to the apical chamber of the Transwell. Cultures were allowed to equilibrate for 24 h after which the HAE cultures were imaged. For each culture, videos of three regions were recorded at ×10 magnification. Images were collected at a frame rate of 0.5 Hz for 60 s on the plane of the mucus gel. Since the secreted mucus tends to accumulate at the edges of the transwells, images were taken centrally to avoid areas of thick mucus. The microsphere tracking data analysis is based on an image processing algorithm that was custom written in MATLAB (78). Briefly, the analysis software computes the *x*-*y* plane trajectories of each fluorescent microsphere in each frame. Using the first and last positions obtained from trajectory data, displacement of microspheres was computed, and transport rate was calculated by dividing the displacement by total time elapsed. Microspheres with transport rates of less than 0.01 μm/s (less than 0.01% of microspheres) were considered immobile and removed from the data set.

### CRISPR/Cas9-mediated knockout of specific mucin glycoproteins in HAE.

To select regions for CRISPR/Cas9-mediated knockout, MUC1 (ENSG00000185499) was analyzed using Ensembl (79). Guide RNA sites were selected based on favorable targeting, Doench, and Xu scores. Putative guides were ordered from IDT with flanking restriction sites for cloning into the plentiCRISPRv2 backbone (80), with enhanced GFP (eGFP) replacing puromycin selection. The final guide targets the region from position 155187791 to 155187813 on chromosome 1 with a WTSI Genome Editing ID of 915343298. To generate negative-control gRNA sequences with no matching sequence in the genome (nontargeting control), we generated 10,000 random sequences of 20 nucleotides and analyzed these candidates using BLAST (81) to characterize their percent identity with the hg19 reference genome. We chose the gRNA sequences with the lowest percent identity, i.e., lowest probability of a sequence match in the genome, as the negative controls. To improve the confidence of our hits, we repeated this process at a wide range of coverage thresholds (99 to 70%) and chose top-ranked candidates consistently ranked among top ones (average rank). We used the following control sequence, which was validated as described above: 5′ CGA CTA CCA GAG CTA ACT CA 3′.

Lentiviral stocks were generated by cotransfection of 1 μg plentiCRISPRv2 (a gift from Feng Zhang) (Addgene plasmid no. 52961; http://n2t.net/addgene:52961; RRID:Addgene_52961), 0.2 μg pCMV-VSV-G (a gift from Bob Weinberg) (Addgene plasmid no. 8454; http://n2t.net/addgene:8454; RRID:Addgene_8454) (82), and 0.7 μg psPAX2 (a gift from Didier Trono) (Addgene plasmid no. 12260; http://n2t.net/addgene:12260; RRID:Addgene_12260) into HEK293T cells with X-tremeGENE HP (no. XTGHP-RO, Roche) in Opti-MEM (no. 31985062, Invitrogen) per the manufacturer’s protocol. Lentivirus-laden supernatant was collected and replaced at 24-h intervals up to 72 h, pooled, and 0.22-μm filtered to remove viable cells and debris.

For target cell transduction, lentivirus-containing supernatant was applied to BCi-NS1.1 (kindly provided by Matthew Walters and Ronald Crystal; maintained as described for HAE above [47]) at 40 to 60% confluence with a final concentration of 20 mM HEPES (no. 15630080, Gibco) and 4 μg/mL Polybrene (no. AB01643, American Bio). Cells were then centrifuged (1,000 × *g* for 1 h at 37°C) and incubated at 37°C overnight. The inoculum was removed and replaced with fresh growth medium. At 60 to 80% confluence, cells were passaged and expanded prior to being sorted for eGFP expression compared to untransduced control cells. Sorted transduced cells were frozen for later use or analyzed with an EnGen mutation detection kit (no. E3321S, New England BioLabs) for on-target gene editing confirmation (forward primer, 5′-AGC ACT TCT CCC CAG TTG TC-3′; reverse primer, 5′-CAG GGA CTG CAC TCA CCA AG-3′). Upon thawing, transduced cells were expanded once before transfer to collagen-coated membranes as with primary HAE. Quantitative confirmation of editing in differentiated HAE was performed by extraction of DNA according to the EnGen mutation detection kit protocol, and the targeted region was amplified using the primers mentioned above. This product was subjected to Sanger sequencing (IDT), and the trace files were analyzed using ICE analysis (Synthego [48]). Target protein depletion in mature, differentiated cultures was confirmed by Western blotting. For histological evaluation, selected cultures were fixed in 4% paraformaldehyde, paraffin embedded, sectioned, and stained with hematoxylin and eosin (H&E) at the New York University Experimental Pathology Research Laboratory and subsequently imaged on a Nikon Eclipse microscope at the University of Maryland Imaging Core.

### Software used and statistical analysis.

FIJI was used to quantify fluorescence intensity and percent antigen-positive area in IF experiments and band intensity in Western blots. Statistical analyses were performed using native GraphPad Prism 8 software. *P* value cutoffs for significance were <0.05, <0.01, <0.001, and <0.0001.
