# Supplementary figures and images for: Membrane-Tethered Mucin 1 Is Stimulated by Interferon and Virus Infection in Multiple Cell Types and Inhibits Influenza A Virus Infection in Human Airway Epithelium
Source: mBio. 2022 Jun 14;13(4):e01055-22. doi: 10.1128/mbio.01055-22 (PMC9426523; doi:10.1128/mbio.01055-22)

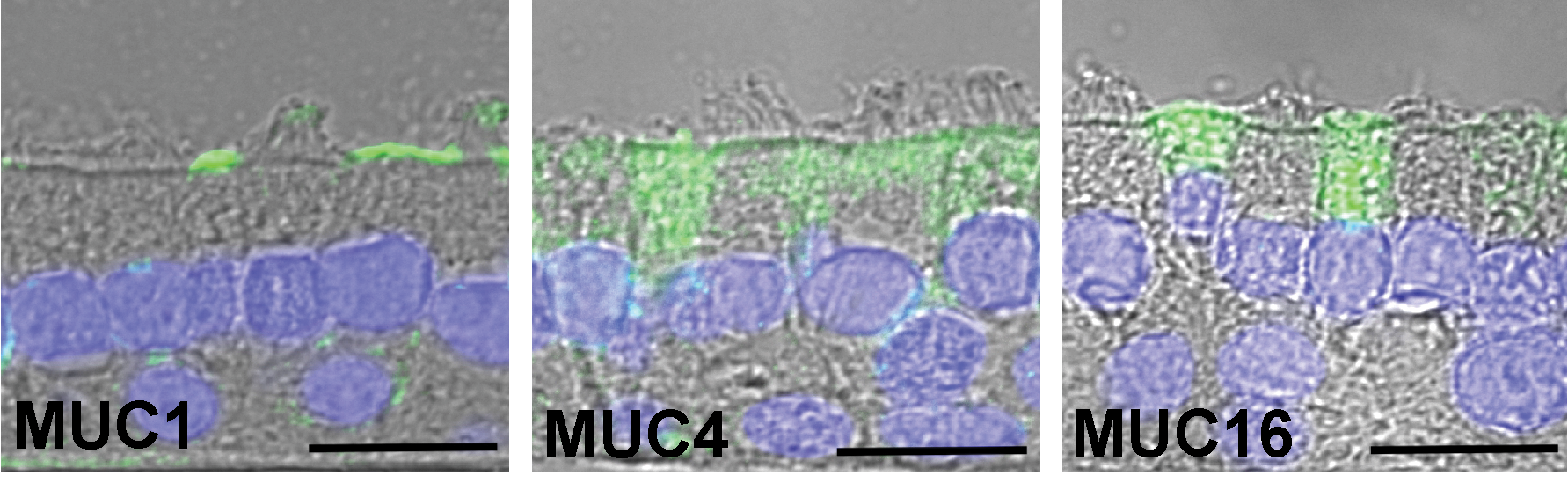

Supplement: FIG S1 [file mbio.01055-22-s0001.tif]

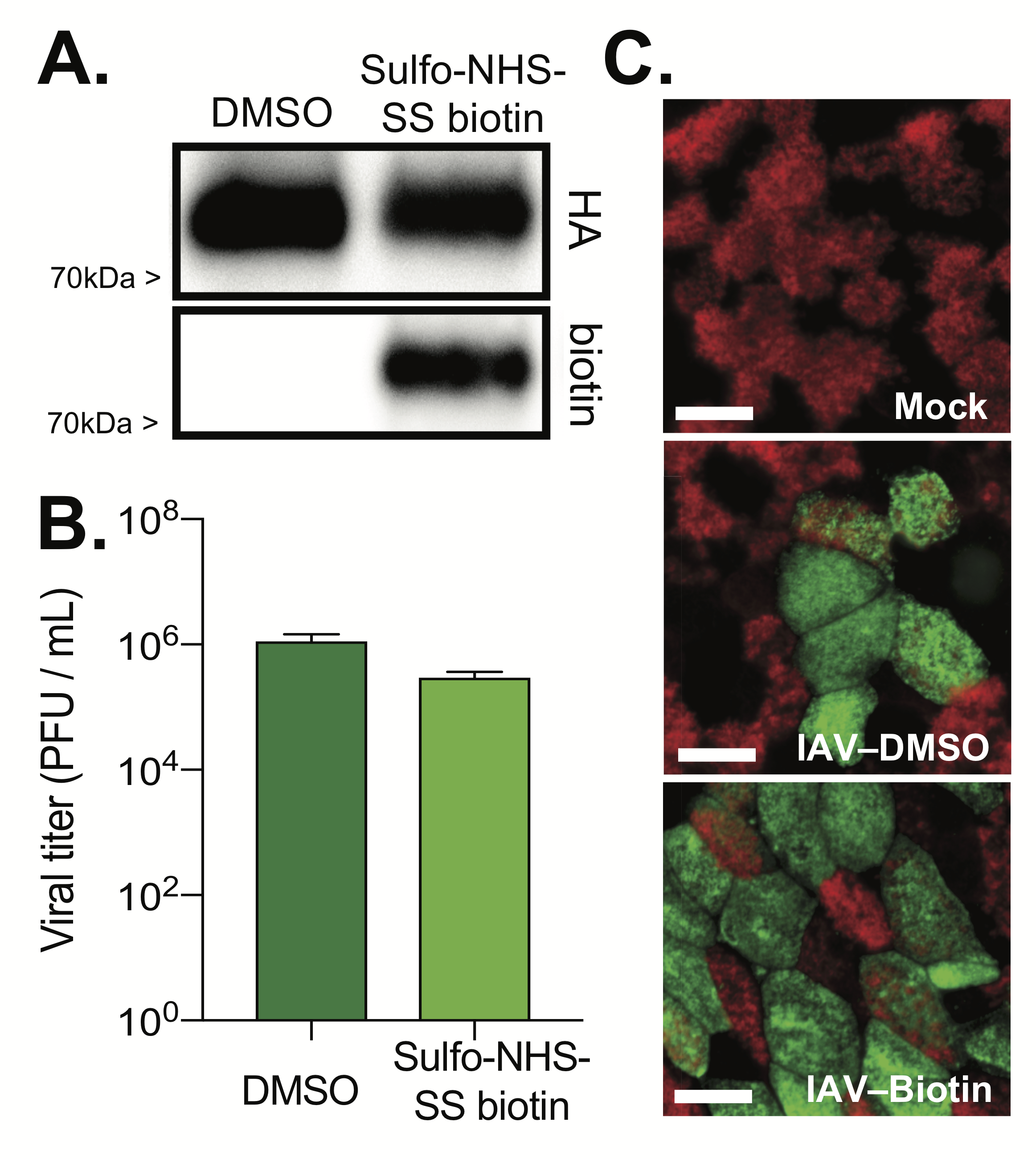

Supplement: FIG S2 [file mbio.01055-22-s0002.tif]

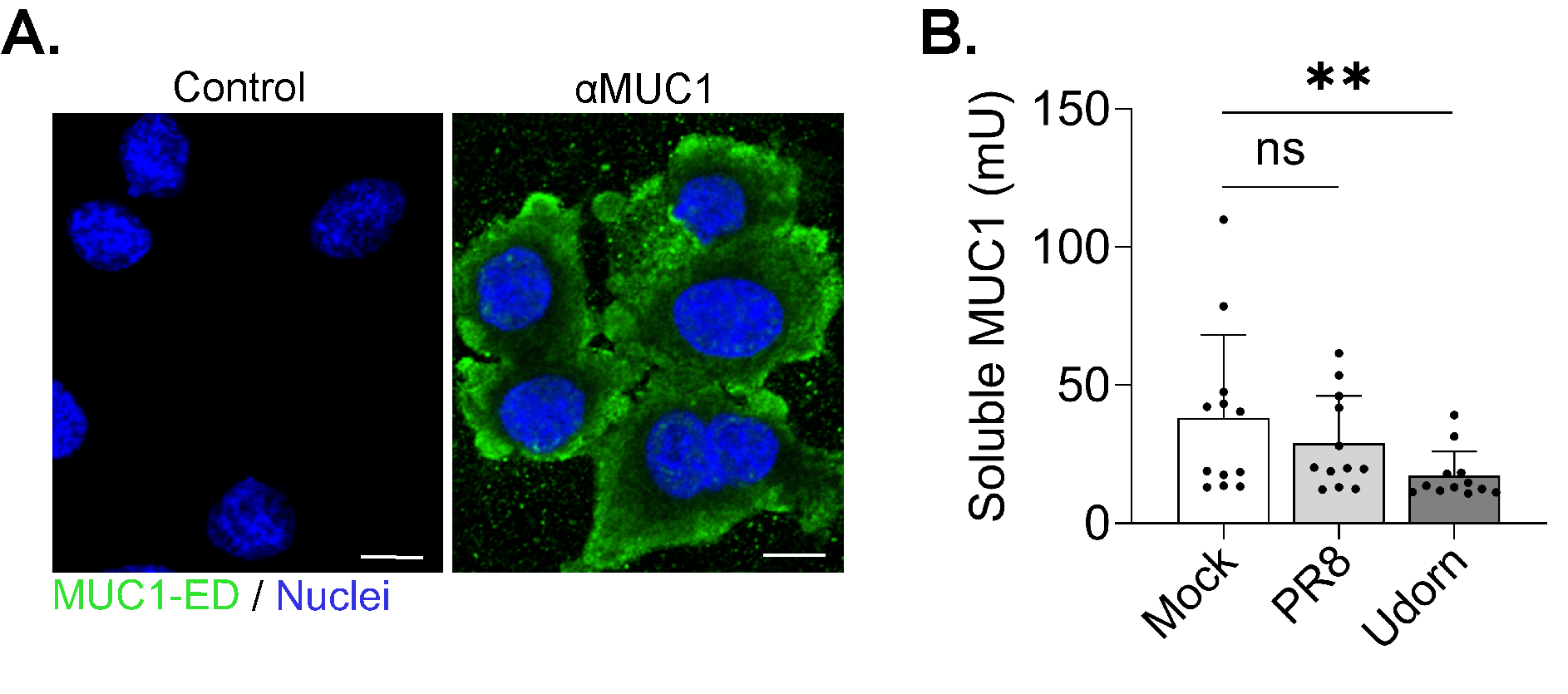

Supplement: FIG S3 [file mbio.01055-22-s0003.tif]

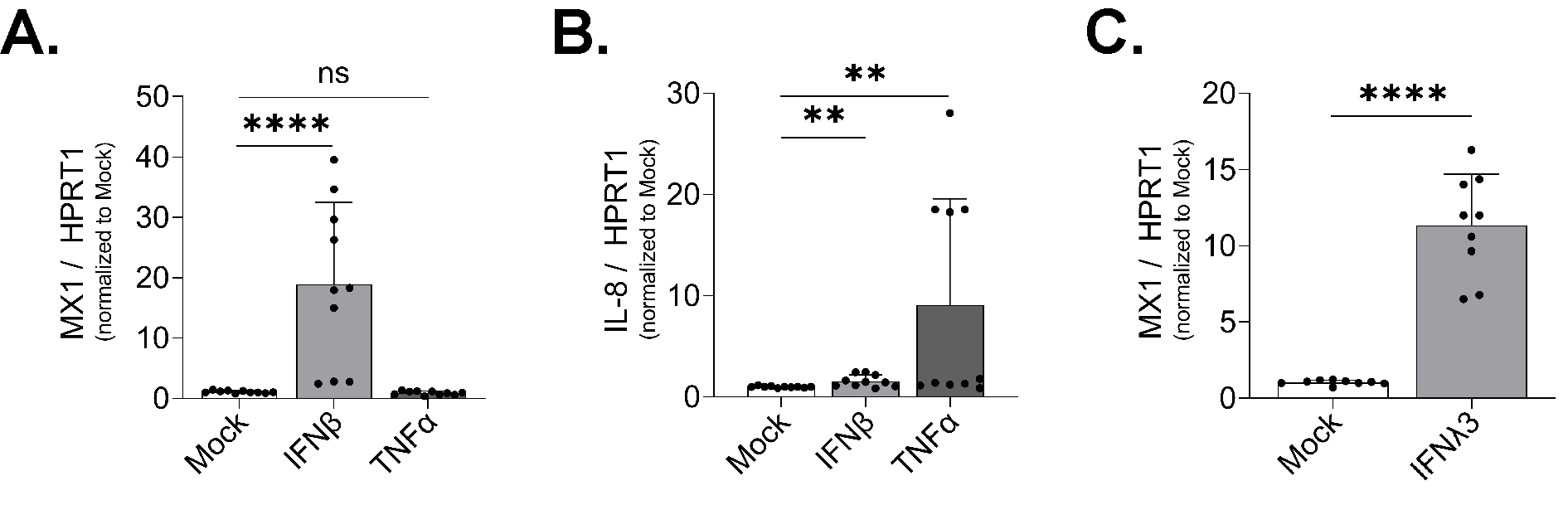

Supplement: FIG S4 [file mbio.01055-22-s0004.tif]

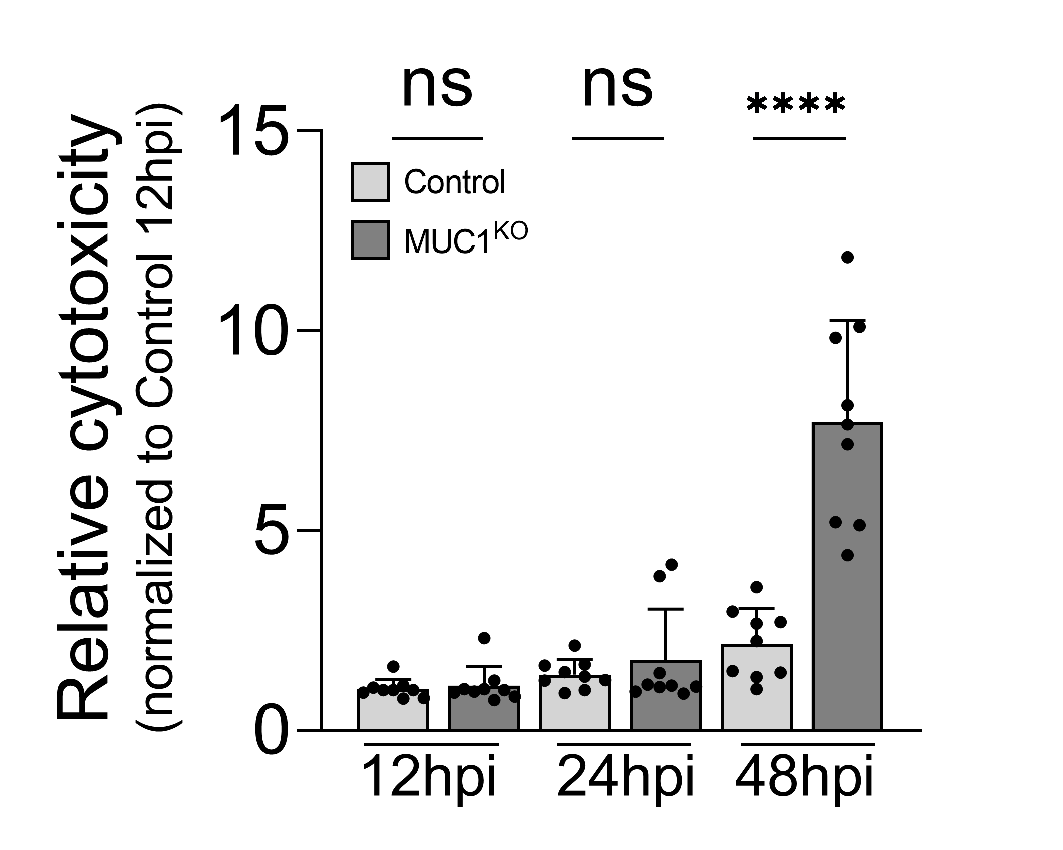

Supplement: FIG S5 [file mbio.01055-22-s0005.tif]
